# Evidence and Potential Mechanisms of Traditional Chinese Medicine for the Adjuvant Treatment of Coronary Heart Disease in Patients with Diabetes Mellitus: A Systematic Review and Meta-Analysis with Trial Sequential Analysis

**DOI:** 10.1155/2022/2545476

**Published:** 2022-08-31

**Authors:** Yu Wei, Qi-You Ding, Chak Yeung, Yi-shan Huang, Bo-xun Zhang, Li-li Zhang, Run-Yu Miao, Sha Di, Lin-Hua Zhao, Xiao-Lin Tong

**Affiliations:** ^1^Institute of Metabolic Diseases, Guang'anmen Hospital, China Academy of Chinese Medical Sciences, Beijing, China; ^2^Graduate College, Beijing University of Traditional Chinese Medicine, Beijing 100029, China; ^3^Department of Endocrinology, Guang'anmen Hospital, China Academy of Chinese Medical Sciences, Beijing 100053, China

## Abstract

Traditional Chinese medicine (TCM) has long been used to treat diabetes mellitus and angina. It has also gained widespread clinical applications in China as a common adjuvant treatment. Although there is high-quality evidence that TCM is effective in regulating glucose and lipid metabolism, the cardiovascular protective effect of TCM in the treatment of diabetes mellitus has not been fully elucidated, especially in patients with both diabetes mellitus and coronary heart disease (CHD). We systematically assessed the efficacy and safety of TCM for the adjuvant treatment of patients with CHD and diabetes mellitus and examined the pharmacological effects and potential mechanisms of TCM medication/herbs on diabetes mellitus with CHD. We found that TCM could improve the control effect of conventional treatment on cardiac function, hemorheology, blood glucose, blood lipid, and inflammation, thus reducing the frequency of angina and the incidence of cardiovascular events and all-cause mortality. These findings indicate that TCM may be used as a complementary approach for patients with diabetes mellitus and CHD. Nevertheless, more rigorously designed randomized controlled trials and long-term evaluations are needed to support these findings.

## 1. Introduction

The global population of patients with diabetes mellitus (DM) is approximately 463 million, which is expected to rise to 700 million by 2040 [1]. As an independent risk factor for coronary heart disease (CHD), DM is considered as “coronary heart disease equivalent” [2], which is associated with increased CHD morbidity and mortality [3]. In patients with DM, the risk of mortality due to CHD is twice that of those who do not have DM [4]. Clinical outcomes and complications following coronary interventions are also worse in such high-risk patients [5]. Several medical resources are spent on DM every year. The total global health expenditure due to DM was estimated to be $673 billion in 2015 [6], with cardiovascular diseases attributable to diabetes accounting for the highest proportion (47.9%) of overall costs [7]. However, the need to control cardiovascular diseases is still unmet. At present, the treatment for DM, especially in those with a high atherosclerotic cardiovascular disease risk, mainly focuses on comprehensively controlling multiple risk factors [8]. However, the complexity of multidrug regimens may induce the chance of nonadherence among patients, while long-term use of high-intensity antiplatelet drugs and lipid-lowering statins may increase the incidence of side effects, such as bleeding, muscle complaints, increased liver or muscle enzymes, or various neurological symptoms [9]. Therefore, clinicians have begun considering traditional Chinese medicine (TCM) as an adjuvant or alternative treatment for DM.

TCM has been widely used to treat diabetes and angina for 1000 years. The holistic and multitarget approaches of TCM have unique advantages in controlling complex diseases, such as DM [10]. In recent years, large-scale clinical trials have demonstrated that TCM effectively reduces hyperglycemia [11–13] and improves vascular inflammation, lowers lipids, and improves cardiac function [14, 15]. A large-sample prospective cohort study also showed that TCM alone is as efficacious as Western medicines in preventing diabetes from being complicated with coronary artery disease [16]. Moreover, patients who used more types of TCM tended to use much less Western medicine recommended by current guidelines [17]. At the same time, some therapeutic mechanisms underlying TCM's potency have been uncovered with the application of modern science and technology.

A number of clinical trials in China have shown that TCM is effective in treating DM or CHD. However, because of the insufficient sample size, short experimental period, and lack of empirical validity of efficacy evaluation in many studies, the results have been questioned and rejected. To date, there are very few systemic analyses that have focused on the comprehensive regulation effect of TCM adjuvant treatment on both DM and CHD. Considering the aforementioned limitations, we selected more credible studies for a systematic review and meta-analysis to comprehensively evaluate the clinical efficacy and safety of TCM for patients with CHD and DM.

## 2. Materials and Methods

We conducted and reported this systematic review following the Preferred Reporting Items for Systematic Reviews and Meta-Analyses (PRISMA) statement [18].

### 2.1. Search Strategy

**Figure 1:**
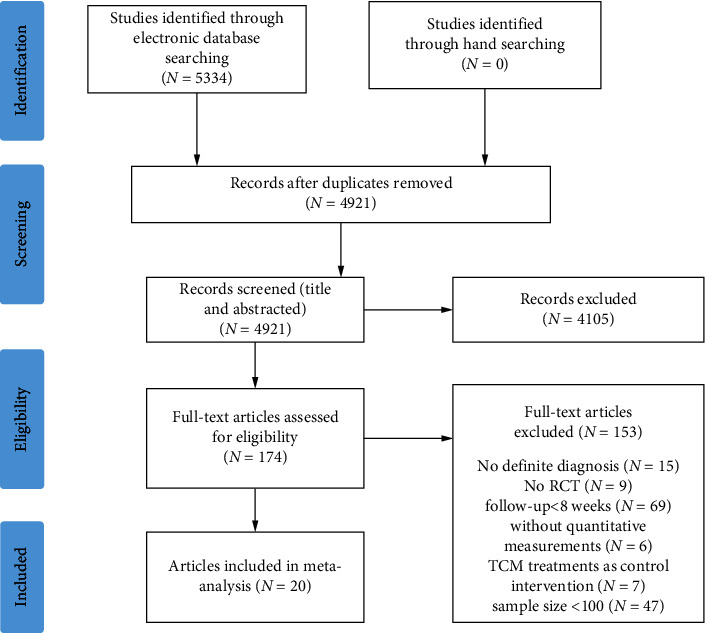
Flow chart of study inclusion.

The first three authors independently searched for papers published from January 2005 to May 2021 on the following databases: the China National Knowledge Internet, Wanfang, VIP databases, the China Biology Medicine, Web of Science, Pubmed, Medline; Embase, International Pharmaceutical Abstracts, and the Cochrane Library. Search terms were as follows: (“coronary heart disease” or “cardiovascular disease” or “angina” or “myocardial ischemia” or “myocardial infarction” or “acute coronary syndrome” or “coronary atherosclerosis”) and (“diabetes” or “diabetes mellitus” or “Xiaoke syndrome”) and (“randomized controlled trial” or “controlled clinical trial” or “random” or “randomly” or “randomized” or “control” or “RCT”) and (“TCM” or “traditional Chinese medicine” or “Chinese medicinal herb” or “Chinese herbal medicine” or “decoction” or “formula” or “prescription” or “Chinese patent medicine” or “Chinese patent drug” or “Chinese herbal compound prescription”). The PRISMA flow chart for the study is presented in Figure 1.

### 2.2. Inclusion Criteria and Study Selection

Clinical studies were included if they satisfied the following criteria: (1) study participants had a definite diagnosis of both DM and CHD and were randomly assigned to receive TCM, contemporary medication, or placebo; (2) the sample size was ≥100; (3) the duration in each study group was ≥8 weeks. Clinical studies with the following features were excluded: (1) nonrandomized trials; (2) the participants had no definite diagnosis; and (3) only symptomatic changes of participants were reported, without objective laboratory measurements or physical examination. As many randomized controlled trials (RCTs) on TCM have been published in Chinese, published reports in both English and Chinese were included. The first two authors (Ding and Wei) independently reviewed all titles and abstracts. If the information was insufficient, the full text was retrieved for further judgment. If the two authors failed to reach a consensus, the senior author (Zhao) made the final judgment.

### 2.3. Quality Assessment

The quality of the included studies was assessed independently by the first two authors (Wei and Ding) as defined by the Cochrane's risk of bias tool [19]. The risk of bias was assigned as low, unclear, or high for the following items: random sequence generation, allocation concealment, blinding of participants and personnel, blinding of outcome assessment, incomplete outcome data, selective reporting, and other bias.

### 2.4. Data Extraction and Analysis

Data extraction was individually performed by the same authors (Wei and Ding) in an unblended standardized manner. In case of discrepancies, the third author (Yeung) was consulted. Relevant reports' information on the author, year, number of participants, intervention (weeks, frequency, and dosage), and passive control condition were extracted and recorded in an excel spreadsheet. The authors of the identified papers were contacted for additional information if necessary. The primary outcomes were all-cause mortality and cardiovascular (CV) events. The frequency of angina contributed to efficacy assessment and was included as a secondary outcome.

Hemorheology indices and cardiac function were also included as secondary outcomes, as abnormalities of these laboratory measurements are associated with CV events. The secondary outcomes also included the levels of fasting blood glucose, postprandial blood glucose (PBG), glycosylated hemoglobin (HbA1c), total cholesterol (TC), triglycerides (TG), low-density lipoprotein cholesterol (LDL-C), high-density lipoprotein cholesterol (HDL-C), C-reaction protein (CRP), interleukin-6 (IL-6), and tumor necrosis factor *α* (TNF-*α*).

RevMan 5.3 (Nordic Cochrane Center, Copenhagen, Denmark) and Stata version 15.0 (Stata Corp., College Station, TX, USA) were used to analyze the data. Continuous outcomes were pooled to find the weighted mean differences (WMDs) accompanied by 95% confidence intervals (CIs), when the outcomes were based on the same scale; otherwise, the standard mean differences (SMDs) were used. Categorical outcomes were pooled to find relative risks (RRs) and were accompanied by 95% CIs. *I*^2^ statistics were used to measure heterogeneity. A fixed-effect model was used if *I*^2^ < 50%; otherwise, the random-effect model was used. Publication bias was explored through a funnel-plot analysis.

### 2.5. Trial Sequential Analysis

In this systematic review and meta-analysis, the curative effect was exaggerated because of random errors, especially when the number of included trials or the total sample size was too small. Thus, the Trial Sequential Analysis (TSA) software version 0.9 Beta (Copenhagen Trial Unit, Copenhagen, Denmark) was used to conduct TSA analysis on the primary outcomes that could be combined and to estimate the meta-analysis sample size and the value strength of the outcome effect. In this study, an overall 5% risk of a type I error was maintained with a power of 80% [20]; the predicted risk reduction was set as 20%, and the relative event rate of the control group was derived from the meta-analysis data.

## 3. Results

### 3.1. Characteristics of the Included Trials

A total of 21 RCTs [21–41] were included in this systematic review. The study by Zhang [34] showed that the HDL levels decreased after treatment, which was contrary to the description of elevated HDL levels. Therefore, this study was excluded from our analysis. Therefore, 20 RCTs were included. One was in English, and the others were in Chinese. The publication years of these studies ranged from 2007 to 2021. The main characteristics of the individual trials are summarized in Table 1. A total of 3565 patients were included; the number of participants in each study ranged from 100 to 591 patients. Only one trial [23] did not indicate the exact number of female and male patients. The overall male to female ratio was approximately 4 : 3. The study period ranged from 8 weeks to 4 years. In all 20 studies, the diagnostic criteria for both DM and CHD were specified. Three studies focused on patients with stable angina pectoris, unstable angina pectoris, or acute coronary syndrome. Chinese medicines used in these studies included both herbal medicines and Chinese patent medicines. The intervention measures in the control group mainly included conventional Western medicines in line with clinical practice and lifestyle modifications, such as exercise and dietary control. Only one study reported that the placebo was delivered to participants in the control group. All 20 studies evaluated multiple outcomes at the end of treatment. Three, two, and the remaining 15 studies reported cardiovascular events, all-cause mortality, and at least two secondary outcome measures, respectively. The specific composition of TCM in the studies is listed in Table 2. Moreover, 12 trials reported adverse events.

### 3.2. Methodological Quality

**Figure 2:**
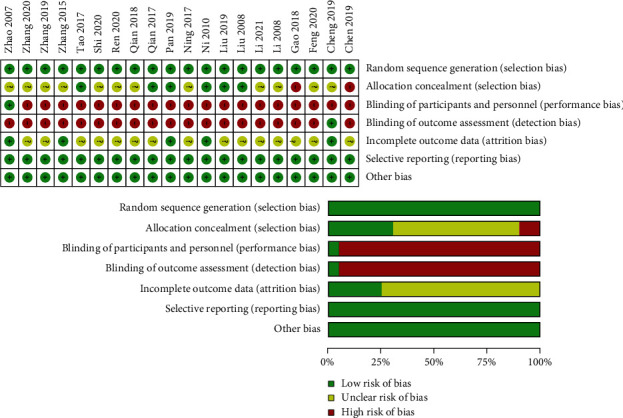
Evaluation of the risk of bias for each included study using the Cochrane Risk of Bias Tool.

All 20 trials claimed that the groups were randomized; nine trials [22–24, 26–28, 30, 32, 35] clearly stated the aforementioned using a random number table, while the others did not specify the method used for random sequence generation. No trials described allocation concealment. Only one trial explicitly mentioned blinding the participants and personnel [36]. The blinding of outcome assessment was not specified in any of the trials. Five studies reported cases of loss to follow-up [23, 28, 33, 35, 36]. Overall, the methodological quality of the included trials was low according to the Cochrane Risk of Bias Tool (Figure 2).

### 3.3. Primary Outcomes

**Figure 3:**
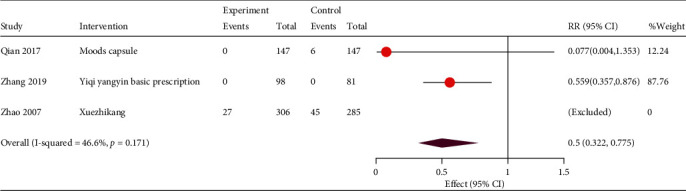
Forest plot of relative risk of all-cause mortality. RR: relative risk; CI: confidence interval.

Three studies reported mortality and cardiovascular events. One study found no death events because of the short follow-up period. Compared to patients in the control group, the beneficial effect of TCM participation on mortality was observed (RR, 0.50; 95% CI, 0.32, 0.78; and *P* = 0.002) with low heterogeneity (*I*^2^ = 47%, *P* = 0.17; Figure 3).

**Figure 4:**
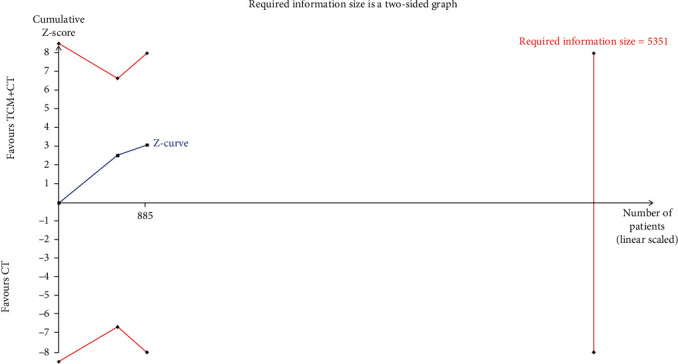
Trial sequential analysis of mortality.

**Figure 5:**
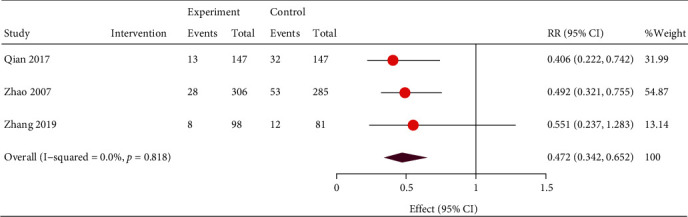
Forest plot of relative risk of incidence of cardiovascular events. RR: relative risk; CI: confidence interval.

**Figure 6:**
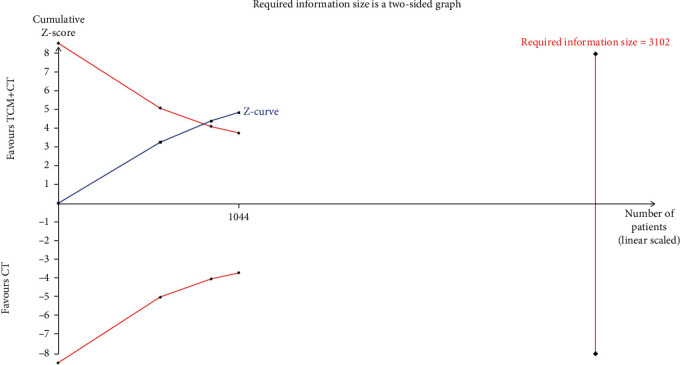
Trial sequential analysis of cardiovascular events.

However, the TSA result indicated a possibility of a false positive conclusion, and more trials should be included to confirm the efficacy (Figure 4). The results were more reliable when it came to reducing cardiovascular events, showing that TCM adjuvant therapy could effectively reduce the incidence of cardiovascular events (RR, 0.47; 95% CI, 0.34, 0.65; and *P* < 0.001) without heterogeneity (*I*^2^ = 0%; *P* = 0.82; Figure 5). The TSA results also supported this conclusion, as shown in Figure 6. The pooled results (*Z* curve, blue lines) crossed both the conventional boundary of benefit (dotted line) and the TSA boundary value curve (full line), indicating that a positive conclusion was reached even though the required information size (*n* = 3102) had not yet been reached (Figure 6).

### 3.4. Secondary Outcomes

#### 3.4.1. Frequency of Angina Pectoris

**Figure 7:**
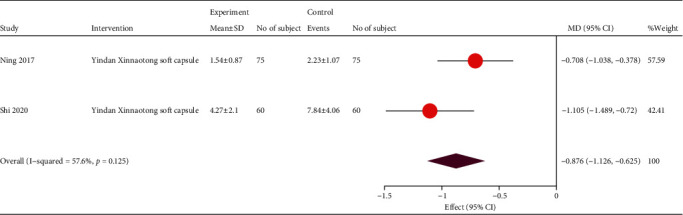
Forest plot of frequency of angina pectoris. MD: mean difference; CI: confidence interval.

Three studies reported data on the change in angina frequency with large heterogeneity. Considering that heterogeneity may be caused by different prescriptions, the pooled effect size analysis was performed according to Ning et al. [29] and Shi et al. [40], with a significant benefit of TCM (MD, -0.88; 95% CI, -1.13, -0.63; and *P* < 0.001), both of which used the Yindan Xinnaotong soft capsules (Figure 7).

#### 3.4.2. Cardiac Function

**Figure 8:**
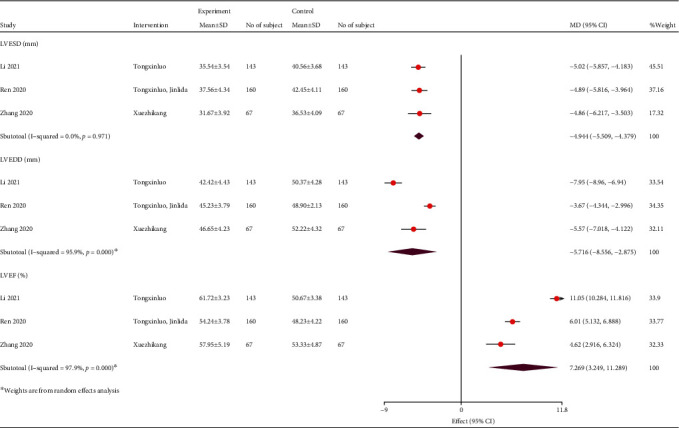
Forest plot of cardiac function. LVEF: left ventricular ejection fraction; LVEDD: left ventricular end diastolic diameter; LVESD: left ventricular end-systolic diameters; MD: mean difference; CI: confidence interval.

Three studies showed an improvement of cardiac function with TCM. Compared to the control group, TCM decreased the left ventricular end-systolic diameters (LVESD) (WMD, -4.94; 95% CI, -5.51, -4.38; *P* = 0.971; and *I*^2^ = 0) and left ventricular end-diastolic diameter (LVEDD) (WMD, -5.06; 95% CI, -5.59, -4.54; *P* < 0.001; and *I*^2^ = 95.9%). TCM significantly increased the left ventricular ejection fraction compared to the control group (WMD, 8.43; 95% CI, 7.89, 8.98; *P* < 0.001; and *I*^2^ = 97.9%; Figure 8).

#### 3.4.3. Hemorheology

**Figure 9:**
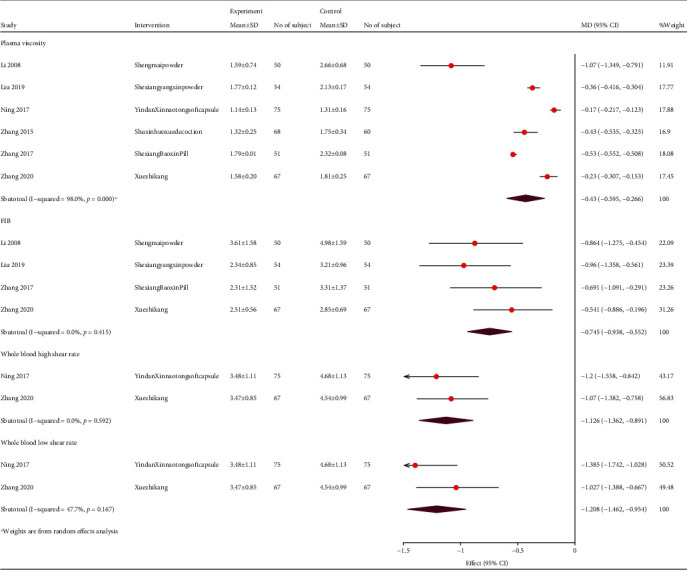
Forest plot of hemorheology. FIB: fibrinogen; MD: mean difference; CI: confidence interval.

Five articles reported changes in the fibrinogen (FIB) levels. The sensitivity analysis found that Ning et al. [29] reported a large effect on heterogeneity; hence, this study was removed from the analysis, and the remaining studies showed that TCM reduced the FIB levels (SMD, -0.75; 95% CI, -0.94, -0.55; *P* = 0.415; and *I*^2^ = 0%). Six articles reported a decrease in plasma viscosity (WMD, -0.44; 95% CI, -0.46, -0.42; *P* < 0.001; and *I*^2^ = 98%). Two articles reported that TCM had an obvious curative effect on whole blood high shear and whole blood low shear (Figure 9).

#### 3.4.4. Systematic Inflammatory Factors

**Figure 10:**
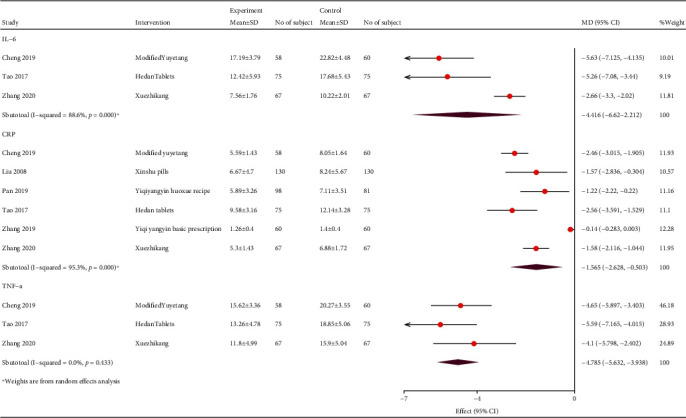
Forest plot of inflammatory factors.

Six studies showed that TCM significantly lowered the TNF-*α* (WMD, -4.79; 95% CI, -5.63, -3.94; *P* = 0.433; and *I*^2^ = 0%), IL-6 (WMD, -3.32; 95% CI, -3.88, -2.76; *P* < 0.001; and *I*^2^ = 88.6%), and CRP (WMD, -0.43; 95% CI, -2.12, -1.04; *P* < 0.001; and *I*^2^ = 95.3%; Figure 10) levels.

#### 3.4.5. Blood Lipid Levels

**Figure 11:**
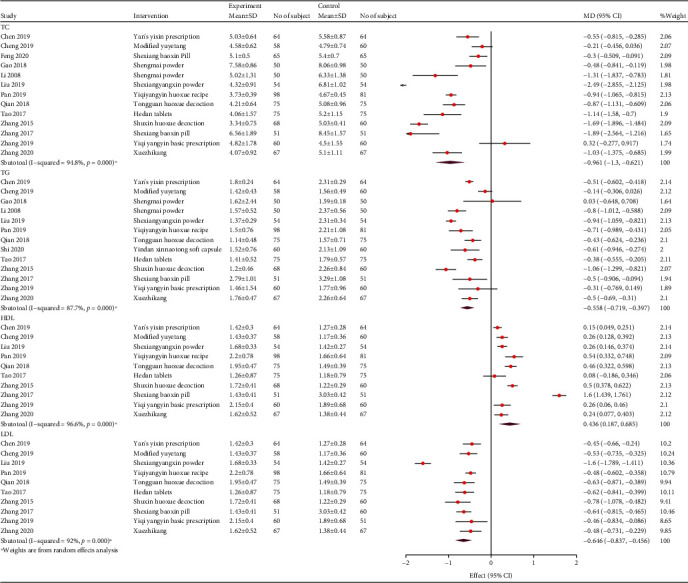
Forest plot of blood lipids.

Fifteen studies reported the blood lipids levels. Eleven studies reported the outcomes of HDL-C. These studies showed that TCM adjuvant therapy improved the HDL-C levels more effectively (WMD, 0.18; 95% CI, 0.15, 0.21; *P* < 0.001; and *I*^2^ = 98.1%). Thirteen studies showed the advantages of TCM adjuvant therapy in reducing TC levels (WMD, -0.83; 95% CI, -0.89, -0.76; *P* < 0.001; and *I*^2^ = 95%). Thirteen studies showed that combined TCM treatment may benefit TG reduction more (WMD, -0.55; 95% CI, -0.6, -0.5; *P* < 0.001; and *I*^2^ = 89.7%). Moreover, 14 studies showed that combined TCM treatment could reduce LDL-C levels more effectively than conventional treatment (WMD, -0.66; 95% CI, -0.7, -0.62; *P* < 0.001; and *I*^2^ = 95.3%; Figure 11). The subgroup analysis of follow-up and prescription is presented in Table 3.

#### 3.4.6. Blood Glucose Levels

**Figure 12:**
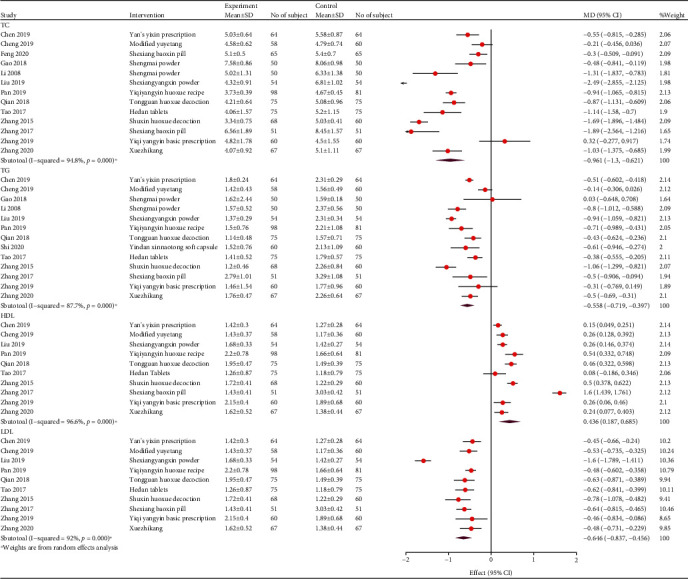
Forest plot of blood glucose levels.

There were nine studies that reported a change in the HbA1c levels, showing statistically significant differences between the conventional and adjuvant TCM treatments (WMD, -1.29; 95% CI, -1.38, -1.2; *P* < 0.001; and *I*^2^ = 96.7%). Eleven studies showed that adjuvant TCM treatment had advantages in controlling FBC (WMD, -0.49; 95% CI, -0.59, -0.4; *P* < 0.001; and *I*^2^ = 80.8%). Further, 11 studies found that TCM intervention reduced the PBG levels more effectively (WMD, -0.98; 95% CI, -1.1, -0.85; *P* < 0.001; and *I*^2^ = 96.4%; Figure 12). The subgroup analysis of follow-up and prescription is presented in Table 3.

### 3.5. Adverse Events

Among the 12 studies that mentioned adverse events, four declared that no significant adverse events occurred with TCM. Gastrointestinal symptoms were reported in the remaining studies; Zhang et al. reported three cases of hypoglycemia, six cases of anorexia, and two cases of headache [41]. Zhao et al. reported two cases of edema, one case of mental-neurological symptoms in the experimental group, and one case of erectile dysfunction in the placebo group [36]. Other adverse events included fever [31], mild headache [25], edema, and itchy skin [21, 30, 31]. There was no statistically significant difference in the incidence of adverse events between the experimental and control groups.

### 3.6. Publication Bias

Funnel plots were drawn in cases where there were >10 articles to examine publication bias regarding the secondary outcomes. Because of the large heterogeneity of the studies, which was difficult to judge by funnel plots alone, Egger's test was performed.

More than 10 studies reported information concerning the TC, TG, LDL, HDL, and 2hPG levels, of which all showed *P* values > 0.05 by Egger's test, indicating no publication bias in the included studies. The funnel plot and Edger's test results for TC, TG, LDL, HDL, and 2hPG are presented in Supplementary materials (available here).

## 4. Discussion

Diabetes combined with CHD increases the incidence of and mortality due to CV events. This review revealed that the use of TCM in addition to conventional treatment showed potential benefits in the treatment of DM with multiple types of CHD. Compared to the existing integrated management of glucose and cardiovascular risk factors, the addition of many TCM prescriptions formed by the delicate compatibility of herbal ingredients showed better efficacy in reducing cardiovascular events. The Standards of Medical Care for Type 2 Diabetes in China 2019 included a dedicated section focusing on TCM treatment [42].

The use of TSA analysis in this paper overcomes the shortcomings of a traditional systematic review or meta-analysis by avoiding hasty conclusions and ignoring the accumulation of random errors. However, it also has certain limitations: it cannot compensate for the defects in the methodological quality of the trial or the errors caused by outcome reporting bias [43].

Articles have compared the efficacy of TCM in the treatment of diabetes and CHD [14], but the outcomes were clinical effectiveness rates, which varied greatly in different studies with no unified standard.

However, combined with the results of previous studies, the use of TCM for the treatment of patients with DM and CHD should be explored further to promote its application.

TCM is characterized by multitargeted effects. The study herein showed beneficial ameliorative effects in the risk factors (blood glucose, lipid, cardiac function, hemorheology, and inflammatory factors) of DM combined with CHD. At present, many Chinese medicines are used to treat cases of DM combined with CHD, but the related clinical studies are insufficient, and the underlying mechanism requires further research. High-quality, randomized, double-blind clinical trials are needed to understand the associated mechanisms.

### 4.1. Limitations

Most of the clinical trials included in this study were high-risk trials. Although all trials claimed to use randomization, only nine described specific randomization methods. Improper randomization often leads to selection bias. At the same time, only one out of 20 studies used a double-blind design, indicating the possibility of potential errors. As the allocation is not concealed, the effect of the intervention may be exaggerated by 30–40% [44]. The reason may be that it is difficult to make a convincing placebo of TCM. This is also a prominent defect of most clinical trials of TCM.

Sensitivity analysis revealed that three studies [25, 29, 36] had a large effect on the heterogeneity of different outcome measures. The work by Zhao et al. [36] was a randomized double-blind trial with intervention for 4 years, which was much longer than the corresponding period in other clinical trials. Interestingly, they reported a clear efficacy on regulating blood lipid indicators [45]. The study suggested that long-term TCM administration may modulate blood lipids better than conventional treatment. The patients included in the study by Li et al. [25] were aged 65–85 (mean age, 74.54) years, which was higher than the corresponding mean ages in other trials. The poor glycemic control, which was possibly attributed to islet cell function, was gradually lost in elderly patients with DM. The patients in the study by Ning et al. [29] received nitrate ester, which can improve hemodynamics and can explain the better control in FIB than other study.

The main sources of heterogeneity were the following: (1) Study protocol designs were not rigorous. (2) The interventions differed greatly in composition, dosage, dosage form, and intervention time. In the theory of TCM, individuals adapted in different formulas for treating DM and CHD are different. Meanwhile, the different dosage forms of TCMs also indicate problems in the quality control of TCMs. (3) Basal interventions differed.

Considering the complicated sources of heterogeneity, we found it difficult to reduce the high heterogeneity through subgroup analysis.

On the one hand, different TCM interventions are main sources of heterogeneity. On the other hand, there are differences in the type of heterogeneity. In addition, although patient baselines were comparable in independent studies, patient conditions, including the severity of CHD, varied between studies.

## 5. Conclusions

Through this systematic review and meta-analysis of adjuvant TCM treatment of CHD in patients with DM, we found that TCM could improve the control effect of conventional treatment on blood glucose, blood lipid, and inflammation, thereby reducing the frequency of angina and the incidence of cardiovascular events and all-cause mortality; this indicates that TCM may be used as a complementary approach to the tertiary prevention of DM. Nevertheless, more rigorously designed RCTs and long-term evaluations are needed to support these findings. The relationship between cardiovascular benefits and TCM control of inflammation, cardiac function, and hemodynamics should be further examined.

## Figures and Tables

**Table 1:** Summary of included studies.

Study	Patient group	Participant (M/W)		Treatment measure		Duration	Outcomes
		Control	Treatment	Control	Treatment		
Chen 2019	T2DM&CHD 128	64 (29/35)	64 (31/33)	CT	Yanshi Yixin decoction+CT	3 m	BG, BL, SAA, MMP-9, TBIL
Cheng 2019	T2DM&CHD 118	60	58	CT	Supplemented Yuye decoction+CT	3 m	BG, BL, IM, SOD, MDA, GSH-Px, 8-ios-PGF2a
Feng 2020	T2DM&CHD 130	65 (33/32)	65 (35/30)	CT	Shexiang Baoxin Pill+CT	3 m	BL
Gao 2018	T2DM&CHD 100	50 (27/23)	50 (24/26)	CT	Shengmai powder+CT	2 m	BG, BL, FGF-21, Ghrelin
Li 2008	DM&CHD 100	50 (33/17)	50 (32/18)	CT	Jiangtang Shengmai decoction+CT	2 m	BG, BL, HI
Li 2021	T2DM&CHD 286	143 (75/68)	143 (76/67)	CT	Tongxinluo capsule+CT	3 m	CF, BL, BG
Liu 2008	T2DM&CHD 260	130 (95/35)	130 (89/41)	CT	Xinshu pill+CT	2 m	BG, IM, DD, LFTs, KFTs
Liu 2019	T2DM&CHD 108	54 (29/25)	54 (28/26)	CT	Shexiang Yangxin powder+CT	3 m	BG, BL, HI
Ni 2010	T2DM&CHD 173	45 (21/24)	128 (55/73)	CT	Qizhi Jiangtang capsule+CT	2 m	BG, dynamic ECG
Ning 2017	T2DM&CHD 150	75 (34/41)	75 (35/40)	CT	Yindan Xinnaotong soft capsule+CT	3 m	HI, angina frequency
Pan 2019	T2DM&SAP 120	60 (29/31)	60 (28/32)	CT	Yiqi Yangyin Huoxue decoction_CT	3 m	BG, BL, CRP, Hcy, UMA
Qian 2017	DM&ACS 294	147 (97/50)	147 (102/45)	CT	Xinyue capsule+CT	6 m	CE, BG, NT-proBNP, CK-MB
Qian 2018	T2DM&CHD 150	75 (43/32)	75 (45/30)	CT	Tongguan Huoxue decoction+Naoxintong capsule+CT	3 m	BG, BL, HMGB1, Omentin-1
Ren 2020	T2DM&CHD 320	160 (85/75)	160 (78/82)	CT	Tongguan Huoxue decoction+Naoxintong capsule+CT	3 m	CF, BG
Shi 2020	T2DM&CHD 120	60 (16/44)	60 (18/42)	CT	Yindan Xinnaotong soft capsule+CT	3 m	Angina frequency, BL
Tao 2017	T2DM&CHD 118	55 (33/22)	63 (43/20)	CT	Hedan pill+CT	6 m	BL, IM, SOD, MDA, NO
Zhang 2015	T2DM&CHD 102	51 (30/21)	51 (28/23)	CT	Shuxin Huoxue decoction+CT	2 m	BL, IM, HI
Zhang 2019	T2DM&SAP 179	81 (43/38)	98 (49/49)	CT	Yiqi Yangyin decoction+CT	3 m	CE, BG, BL, HI, IM
Zhang 2020	DM&CHD 134	67 (36/31)	67 (34/33)	CT	Xuezhikang+CT		BG, BL, HI, IM, CF
Zhao 2007	T2DM&CHD 591	285 (198/87)	306 (226/80)	Placebo+CT	Xuezhikang+CT	4 y	ACM, CE, BL

ACS: acute coronary syndrome; SAP: stable angina pectoris; CT: conventional treatment; ACM: all-cause mortality; CE: cardiovascular events; CF: cardiac function; HI: hemorrheologic indices; IM: inflammatory marks; BG: blood glucose; BL: blood lipid.

**Table 2:** Composition of TCM in the study.

Study	Prescription	Medicine	Article
Chen 2019	Yan's yixin prescription	Dasheng (Radix Codonopsis), Huangqi (Radix Astragali seu Hedysari), Juemingzi (Semen Cassiae), Gegen (Radix Puerariae), Shichangpu (Rhizoma Acori Tatarinowii), Jiangxiang (Lignum Dalbergiae Odoriferae), Danshen (Radix Salviae Miltiorrhizae), Chuanxiong(Rhizoma Ligustici Chuanxiong), Chishao (Radix Paeoniae Rubra), Shenzha (Fructus Crataegi), Fabanxia (Rhizoma Pinelliae Preparatum), Zhiqiao (Fructus Aurantii)	[22]
Cheng 2019	Modified Yuyetang	Shanyao (Rhizoma Dioscoreae), Taizishen (Radix Pseudostellariae), Huangqi (Radix Astragali seu Hedysari), Zhimu (Rhizoma Anemarrhenae), Gegen (Radix Puerariae), Wuweizi (Fructus Schisandrae Chinensis), Tianhuafen (Radix Trichosanthis), Jineijin (Endothelium Corneum Gigeriae Galli), Danshen (Radix Salviae Miltiorrhizae), Sanqi (Radix Notoginseng), Chuanxiong (Rhizoma Ligustici Chuanxiong), Hongqu (fermentum rubrum), Fabanxia (Rhizoma Pinelliae Preparatum), Chenpi (Pericarpium Citri Reticulatae), Gancao (Radix Glycyrrhizae)	[23]
Feng 2020	Shexiang Baoxin Pill	Shexiang (Moschus), Renshen (Radix Ginseng), Niuhuang (Calculus Bovis), Rougui (Cortex Cinnamomi), Anxixiang (Benzoinum), Chansu (Venenum Bufonis), Bingpian (Borneolum Syntheticum)	[46]
Gao 2018	Shengmai powder	Gancao (Radix Glycyrrhizae), Wuweizi (Fructus Schisandrae Chinensis), Huangqi (Radix Astragali seu Hedysari), Renshen (Radix Ginseng), Chuanxiong(Rhizoma Ligustici Chuanxiong), Maidong (Radix Ophiopogonis), Gegen (Radix Puerariae), Danshen (Radix Salviae Miltiorrhizae), Sanqi (Radix Notoginseng)	[24]
Li 2008	Shengmai powder	Renshen (Radix Ginseng), Huangqi (Radix Astragali seu Hedysari), Shanyao (Rhizoma Dioscoreae), Cangzhu (Rhizoma Atractylodis), Xuanshen (Radix Scrophulariae), Danggui (Radix Angelicae Sinensis), Shudi (Radix Rehmanniae Preparata), Maidong (Radix Ophiopogonis), Wuweizi (Fructus Schisandrae Chinensis), Danshen (Radix Salviae Miltiorrhizae), Chuanxiong (Rhizoma Ligustici Chuanxiong), Chishao (Radix Paeoniae Rubra), Yanhusuo (Rhizoma Corydalis), Gancao (Radix Glycyrrhizae)	[25]
Li 2021	Tongxinluo	Renshen (Radix Ginseng), Shuizhi (Hirudo), Quanxie (Scorpio), Chishao (Radix Paeoniae Rubra), Chantui (Periostracum Cicadae), Tubiechong (Eupolyphaga Seu Steleophaga), Wugong (Scolopendra), Tanxiang (Lignum Santali Albi)	[47]
Liu 2008	Xinshu pills	Huangqi (Radix Astragali seu Hedysari), Danshen (Radix Salviae Miltiorrhizae), Gegen (Radix Puerariae), Dasheng (Radix Codonopsis), Shuizhi (Hirudo), Jiezi (Semen Sinapis Albae), Huangjing (Rhizoma Polygonati), Maidong (Radix Ophiopogonis), Wuweizi (Fructus Schisandrae Chinensis)	[26]
Liu 2019	Shexiangyangxin powder	Danshen (Radix Salviae Miltiorrhizae), Xiebai (Bulbus Allii Macrostemonis), Maidong (Radix Ophiopogonis), Chishao (Radix Paeoniae Rubra), Renshen (Radix Ginseng), Guizhi (Ramulus Cinnamomi), Wuweizi (Fructus Schisandrae Chinensis), Sharen (Fructus Amomi Villosi), Tanxiang (Lignum Santali Albi), Bingpian (Borneolum Syntheticum), Shexiang (Moschus)	[27]
Ni 2010	Qizhi jiangtang capsule	Huangqi (Radix Astragali seu Hedysari), Shudi (Radix Rehmanniae Preparata), Huangjing (Rhizoma Polygonati), Shuizhi (Hirudo)	[48]
Ning 2017	Yindan Xinnaotong soft capsule	Yinxingye (Folium Ginkgo), Danshen (Radix Salviae Miltiorrhizae), Xixin (Herba Asari), Jiaogulan (Gynostemma pentaphylla), Shanzha (Fructus Crataegi), Sanqi (Radix Notoginseng), Aiye (Folium Artemisiae Argyi)	[29]
Pan 2019	Yiqiyangyin huoxue recipe	Taizishen (Radix Pseudostellariae), Huangqi (Radix Astragali seu Hedysari), Danggui (Radix Angelicae Sinensis), Shudi (Radix Rehmanniae Preparata), Danshen (Radix Salviae Miltiorrhizae), Guijianyu (Ramuli euonymi), Xuanshen (Radix Scrophulariae), Danpi (Cortex Moutan Radicis), Shanzhuyu (Fructus Corni), Gegen (Radix Puerariae), Sanqi (Radix Notoginseng)	[35]
Qian 2017	Moods capsule	Panax quinquefolium saponin	[32]
Qian 2018	Tongguan huoxue decoction	Shudi (Radix Rehmanniae Preparata), Xiyangshen (Radix Panacis Quinquefolii), Huangjing (Rhizoma Polygonati), Huangqi (Radix Astragali seu Hedysari), Maidong (Radix Ophiopogonis), Gualou (Fructus Trichosanthis), Honghua (Flos Carthami), Chuanxiong (Rhizoma Ligustici Chuanxiong), Dilong (Lumbricus), Chaihu (Radix Bupleuri), Gancao (Radix Glycyrrhizae)	[21]
Ren 2020	Tongxinluo, Jinlida	Renshen (Radix Ginseng), Shuizhi (Hirudo), Quanxie (Scorpio), Chishao (Radix Paeoniae Rubra), Chantui (Periostracum Cicadae), Tubiechong (Eupolyphaga Seu Steleophaga), Wugong (Scolopendra), Tanxiang (Lignum Santali Albi), Cangzhu (Rhizoma Atractylodis), Baizhu (Rhizoma Atractylodis Macrocephalae), Gegen (Radix Puerariae), Yuzhu (Rhizoma Polygonati Odorati), Fuling (Poria)	[47, 49]
Shi 2020	Yindan Xinnaotong soft capsule	Yinxingye (Folium Ginkgo), Danshen (Radix Salviae Miltiorrhizae), Xixin (Herba Asari), Jiaogulan (Gynostemma pentaphylla), Shanzha (Fructus Crataegi),Sanqi (Radix Notoginseng), Aiye (Folium Artemisiae Argyi)	[39]
Tao 2017	Hedan tablets	Heye (Folium Nelumbinis), Danshen (Radix Salviae Miltiorrhizae), Shanzha (Fructus Crataegi), Fanxieye (Folium Sennae), Buguzhi (Fructus Psoraleae)	[30]
Zhang 2015	Shuxin huoxue decoction	Huangqi (Radix Astragali seu Hedysari), Maidong (Radix Ophiopogonis), Danshen (Radix Salviae Miltiorrhizae), Huangjing (Rhizoma Polygonati), Shuizhi (Hirudo), Jiezi (Semen Sinapis Albae), Dasheng (Radix Codonopsis), Wuweizi (Fructus Schisandrae Chinensis)	[33]
Zhang 2017	Shexiang Baoxin Pill	Danshen (Radix Salviae Miltiorrhizae), Xiebai (Bulbus Allii Macrostemonis), Maidong (Radix Ophiopogonis), Chishao (Radix Paeoniae Rubra), Renshen (Radix Ginseng), Guizhi (Ramulus Cinnamomi), Wuweizi (Fructus Schisandrae Chinensis), Sharen (Fructus Amomi Villosi), Tanxiang (Lignum Santali Albi), Bingpian (Borneolum Syntheticum), Shexiang (Moschus)	[34]
Zhang 2019	Yiqi yangyin basic prescription	Taizishen (Radix Pseudostellariae), Huangqi (Radix Astragali seu Hedysari), Gegen (Radix Puerariae), Shudi (Radix Rehmanniae Preparata), Danggui (Radix Angelicae Sinensis), Guijianyu (Ramuli euonymi), Danpi (Cortex Moutan Radicis), Shanzhuyu (Fructus Corni)	[31]
Zhang 2020, Zhao 2007	Xuezhikang	Hongqu (fermentum rubrum)	[41, 36]

**Table 3:** The subgroup analysis of blood lipids and blood glucose.

Outcomes	No. of studies	No. of experiment	No. of control	MD	95% CI	*P* value	*I* ^2^ (%)
TC							
Total	13	1141	1097	-1.03	-1.46, -0.60	<0.001	95.2
*Subgroup: follow-up*							
2 months	3	168	160	-1.41	-2.67, -0.15	<0.001	96.1
3 months	8	541	526	-1.02	-1.61,-0.42	<0.001	95.1
6 months	1	75	75	-0.83	-1.16, -0.49		
4 years	1	285	306	-0.29	-0.45, -0.13		
*Subgroup: prescription*							
Xuezhikang	2	373	352	-0.63	-1.34, 0.07	<0.001	92.1
TG							
Total	13	1141	1097	-0.93	-1.32, -0.53	<0.001	94.3
*Subgroup: follow-up*							
2 months	3	168	160	-1.02	-2.06, -0.03	<0.001	94.8
3 months	8	541	526	-1.03	-1.54, -0.53	<0.001	93.3
6 months	1	75	75	-0.697	-1.026, -0.367		
4 years	1	285	306	-0.062	-0.223, 0.099		
*Subgroup: prescription*							
Xuezhikang	2	373	352	-0.46	-1.27,0.35	<0.001	94.2
HDL							
Total	11	1068	1024	0.72	0.41, 1.02	<0.001	90.7
*Subgroup: follow-up*							
2 months	1	68	60	1.39	1.01, 1.78		
3 months	8	619	604	0.8	0.54, 1.06	<0.001	79.5
6 months	1	75	75	0.096	-0.224, 0.417		
4 years	1	306	285	0.141	-0.02,0.3		
*Subgroup:prescription*							
Xuezhikang	2	373	352	0.29	-0.06, 0.63	0.066	70.5
LDL							
Total	14	1243	1199	-1.20	-1.61, -0.79	<0.001	95.2
*Subgroup: follow-up*							
2 months	2	110	118	-0.88	-1.15, -0.6	0.711	0
3 months	10	744	729	-1.38	-1.95, -0.81	<0.001	95.8
6 months	1	75	75	-0.898	-1.23, -0.56		
4 years	1	306	285	-0.459	-0.62, -0.3		
*Subgroup: prescription*							
Xuezhikang	2	373	352	-0.49	-0.64, -0.34	0.337	0
FBG							
Total	13	1143	1045	-0.5	-0.67, -0.33	<0.001	72.4
*Subgroup: follow-up*							
2 months	4	358	275	-0.44	-0.73, -0.15	0.04	64.9
3 months	8	725	710	-0.49	-0.7, -0.27	<0.001	76.5
6 months	1	60	60	-0.886	-1.26, -0.51		
*Subgroup: prescription*							
Tongxinluo	2	303	303	-0.43	-0.86, -0.01	0.009	85.2
HbA1c							
Total	9	679	664	-1.159	-1.85, -0.47	<0.001	96.9
*Subgroup: follow-up*							
3 months	8	619	604	-0.95	-1.63, -0.28	<0.001	96.6
6 months	1	60	60	-2.85	-3.36,-2,3		
2hPG							
Total	10	948	850	-0.58	-1, -0.16	<0.001	94.5
*Subgroup: follow-up*							
2 months	4	358	275	-0.42	-0.72, -0.11	0.02	68.4
3 months	6	575	590	-0.69	-1.37, -0.01	<0.001	96.6
*Subgroup: prescription*							
Tongxinluo	2	303	303	-1.42	-3.33, 0.49	<0.001	99

## Data Availability

The data used to support the findings of this study are included within the article.

## Supplementary Materials

Supplementary Materials Supplementary Materials The metaregression analysis of sample, publication year, and sensitivity analysis are available in supplementary materials.
