# Supplementary material for: Evidence and Potential Mechanisms of Traditional Chinese Medicine for the Adjuvant Treatment of Coronary Heart Disease in Patients with Diabetes Mellitus: A Systematic Review and Meta-Analysis with Trial Sequential Analysis
Source: J Diabetes Res. 2022 Aug 31;2022:2545476. doi: 10.1155/2022/2545476 (PMC9453102; doi:10.1155/2022/2545476)

**1．Meta-regression analysis**

**1.1 meta-regression of sample**

**
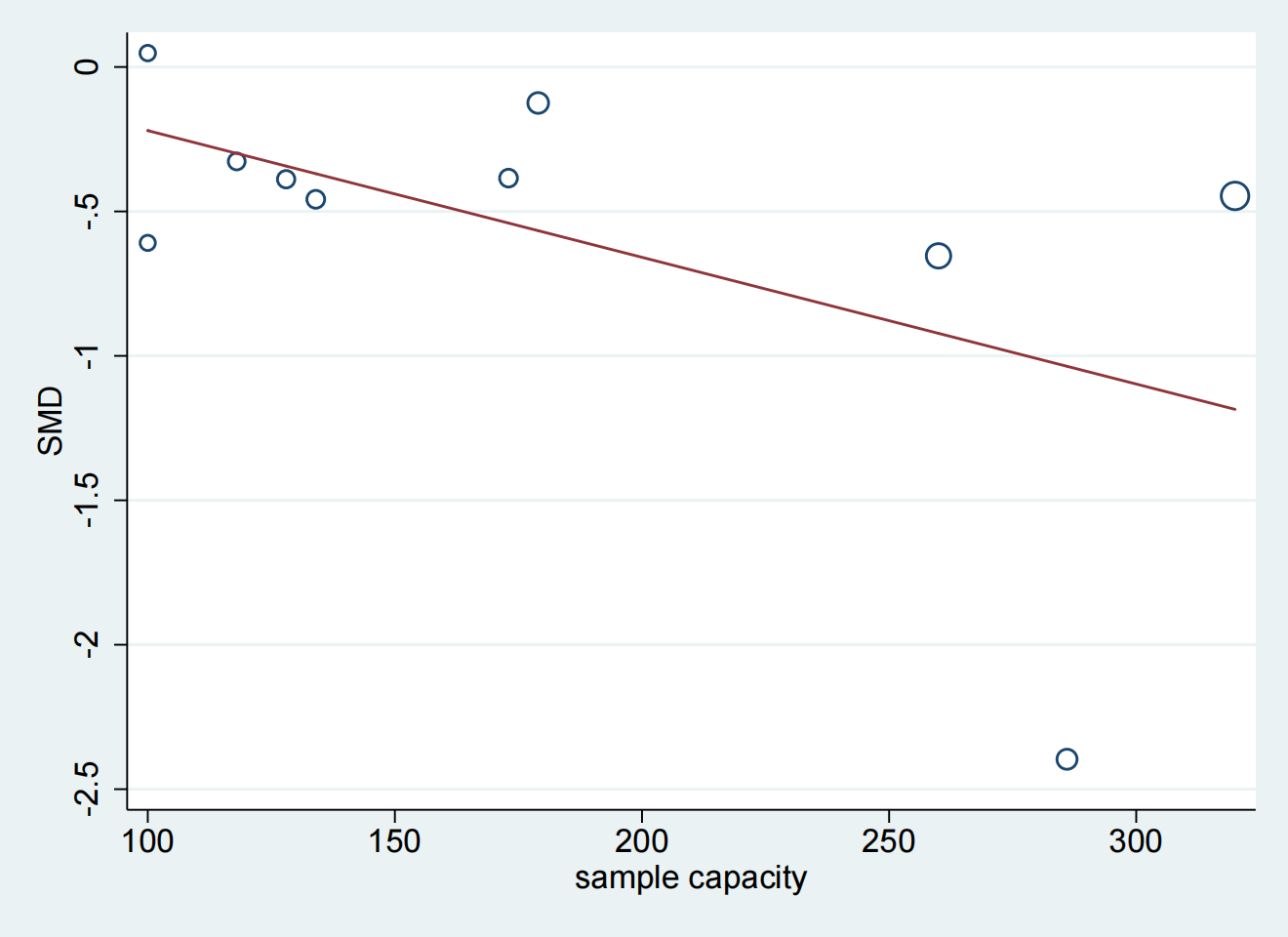
**

**Figure 1 Regression of sample on 2hPG**

**
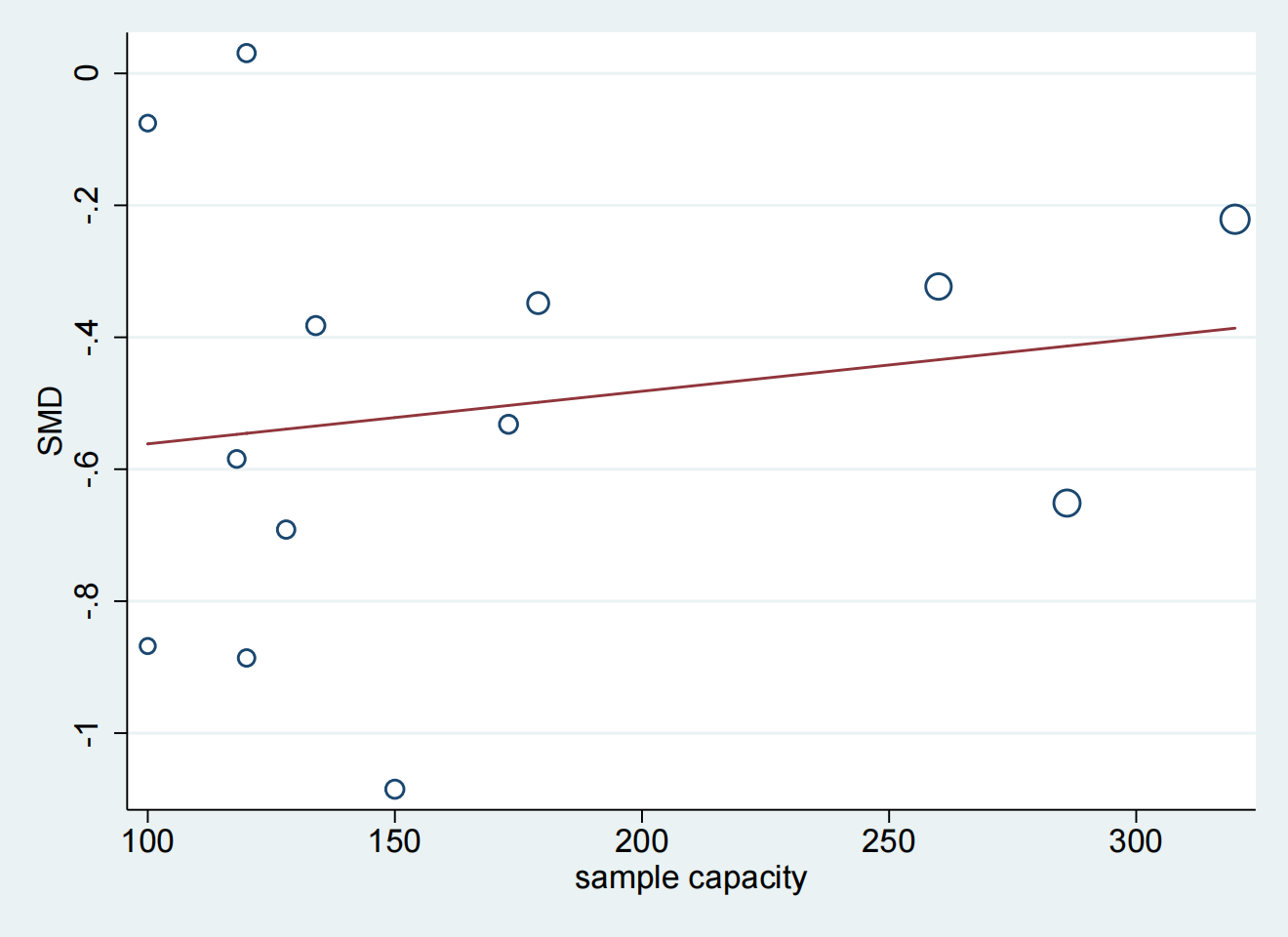
**

**Figure 2 Regression of sample on FPG**

**
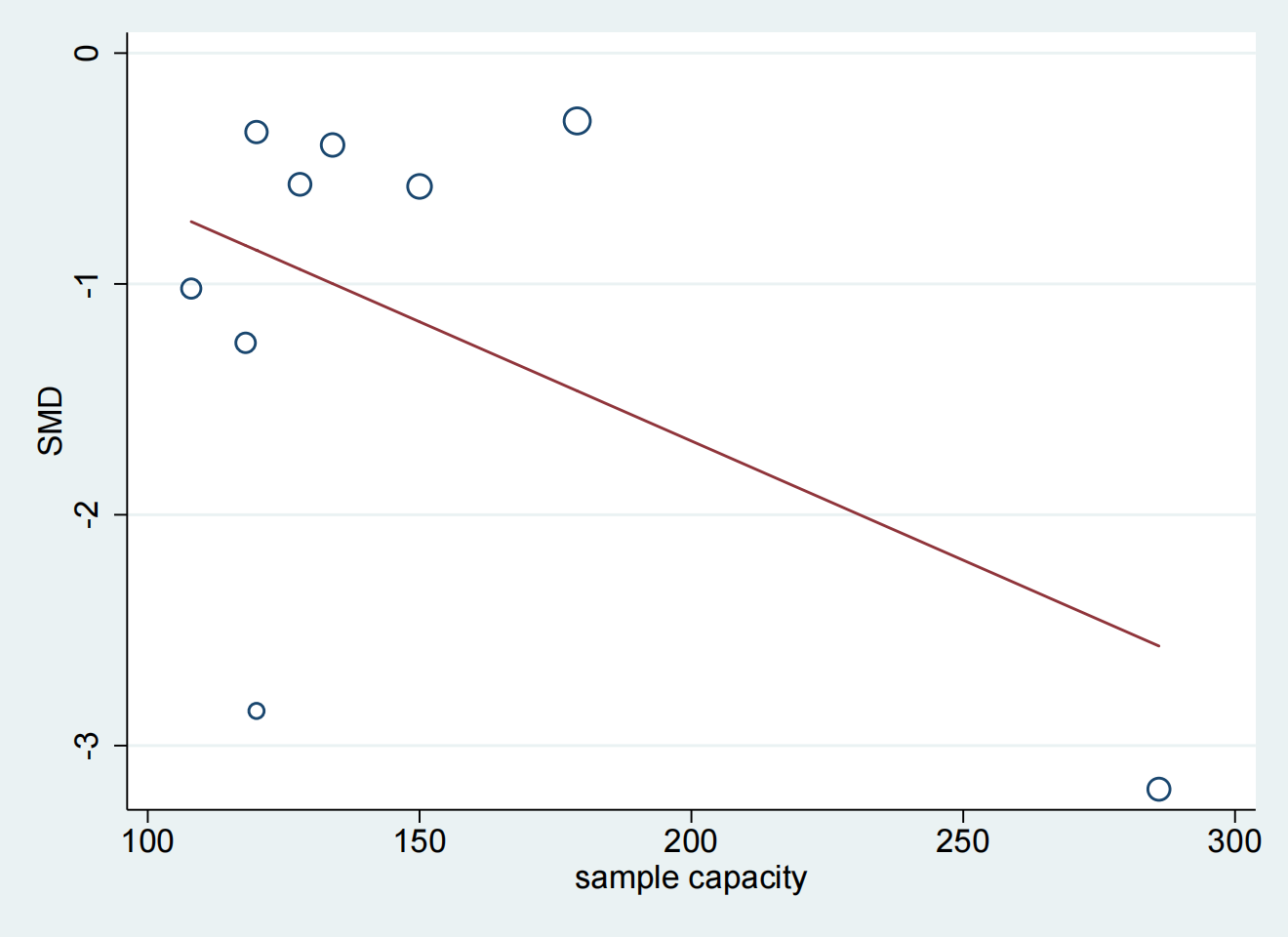
**

**Figure 3 Regression of sample on HbA1c**

**
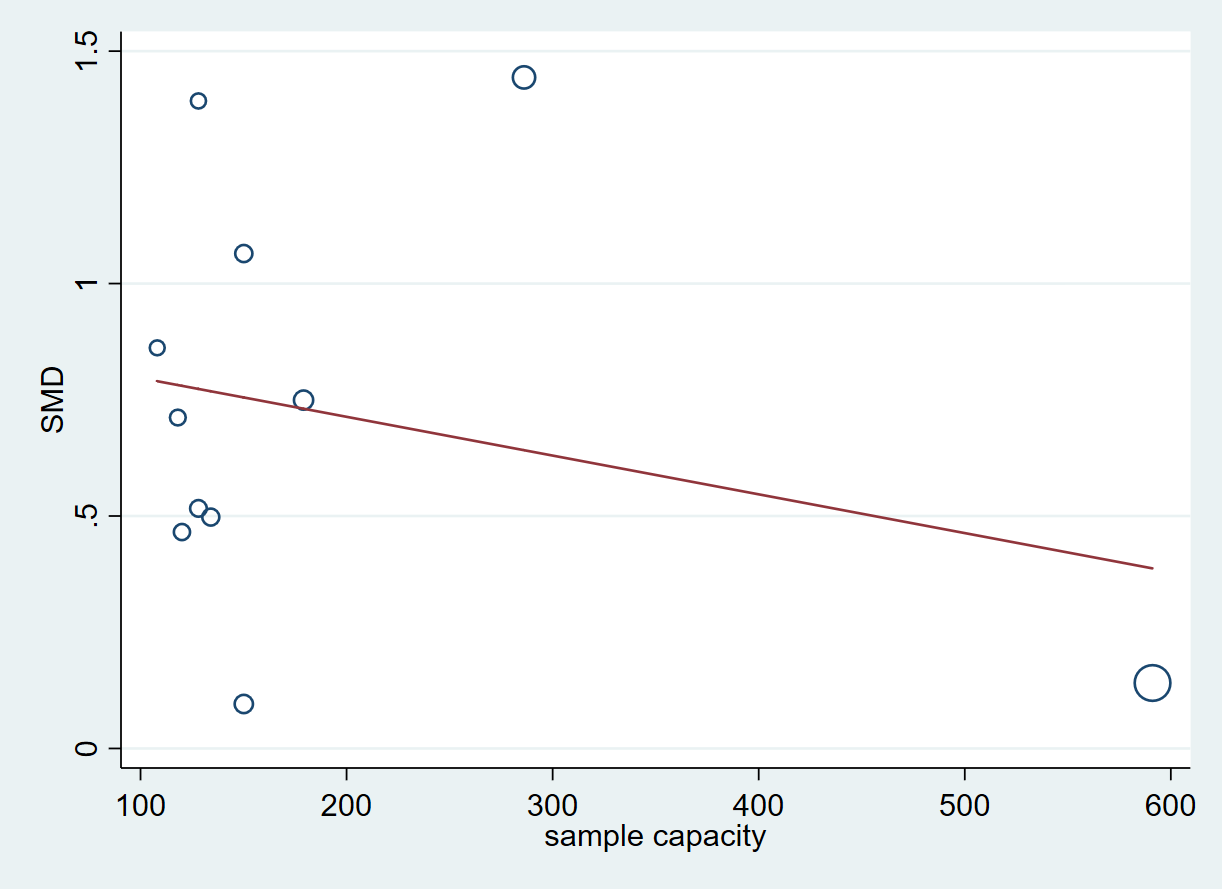
**

**Figure 4 Regression of sample on HDL**

**
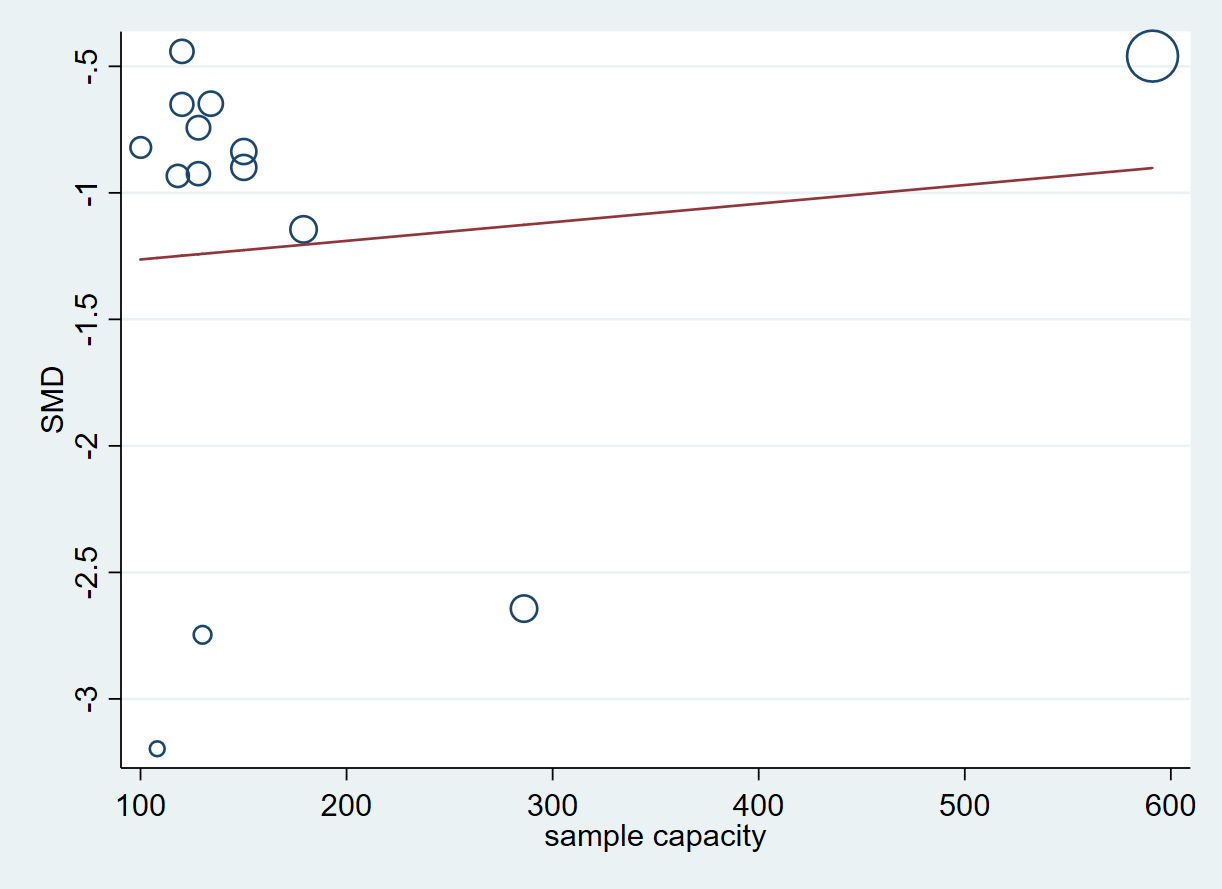
**

**Figure 5 Regression of sample on LDL**

**
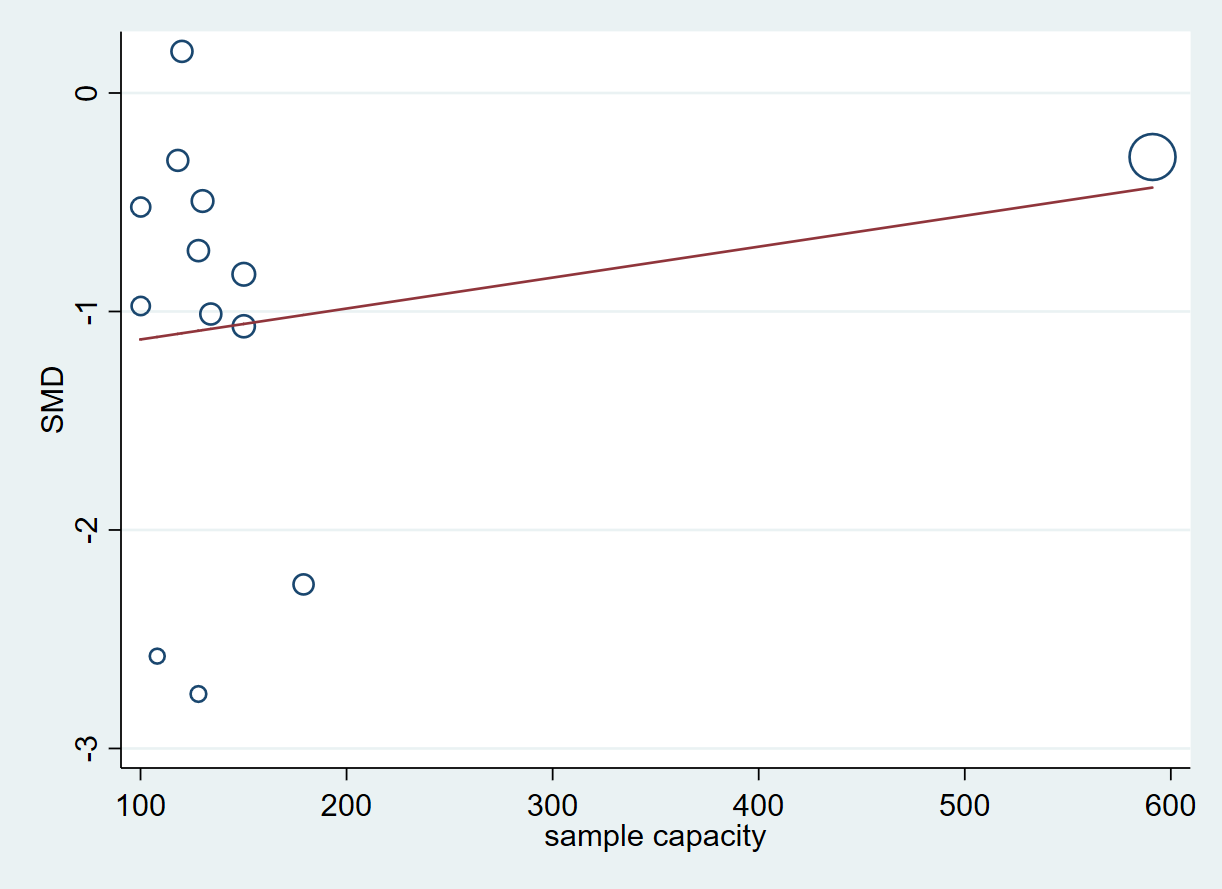
**

**Figure 6 Regression of sample on TC**

**
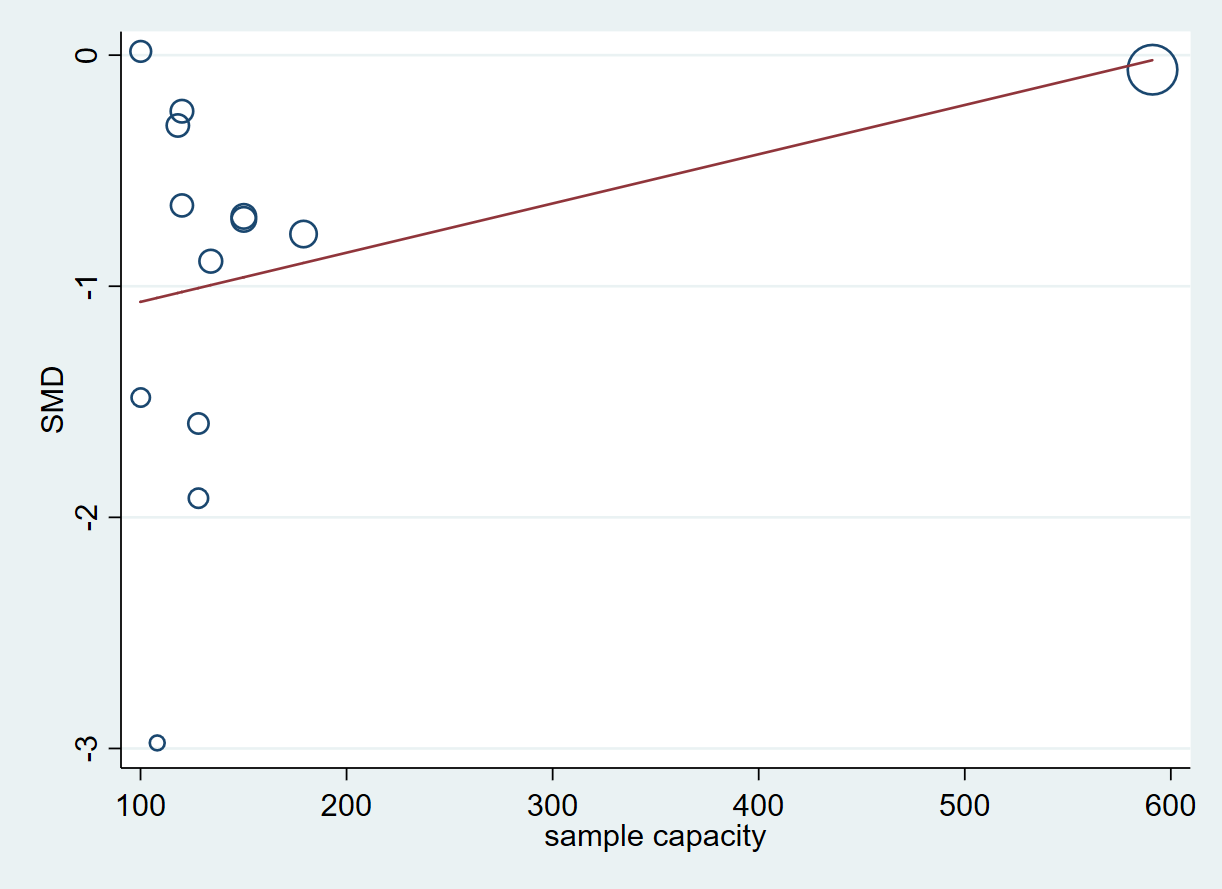
**

**Figure 7 Regression of sample on TG**

**1.2 Regression of publication year**

**
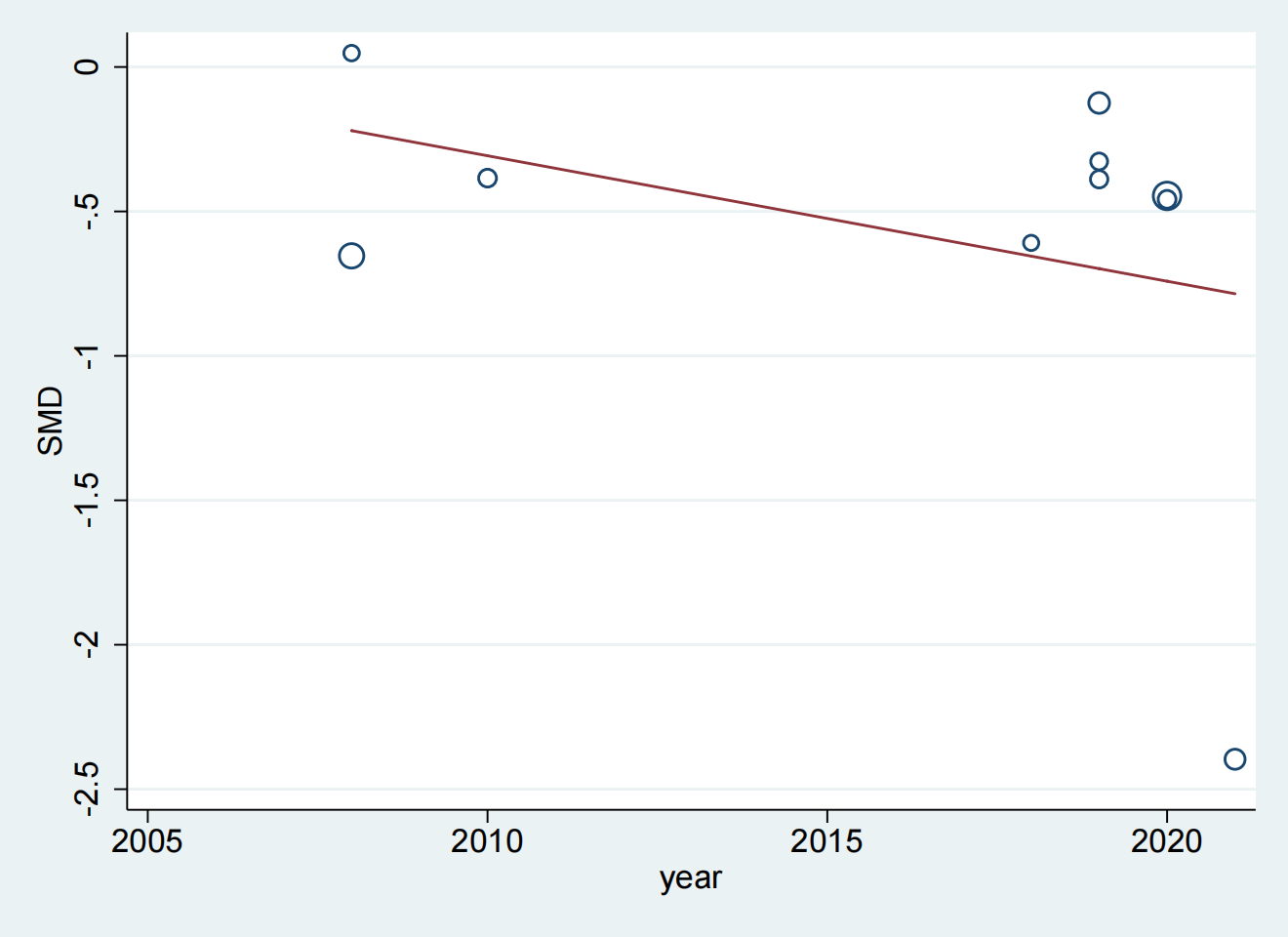
**

**Figure 8 Regression of year on 2hPG**

**
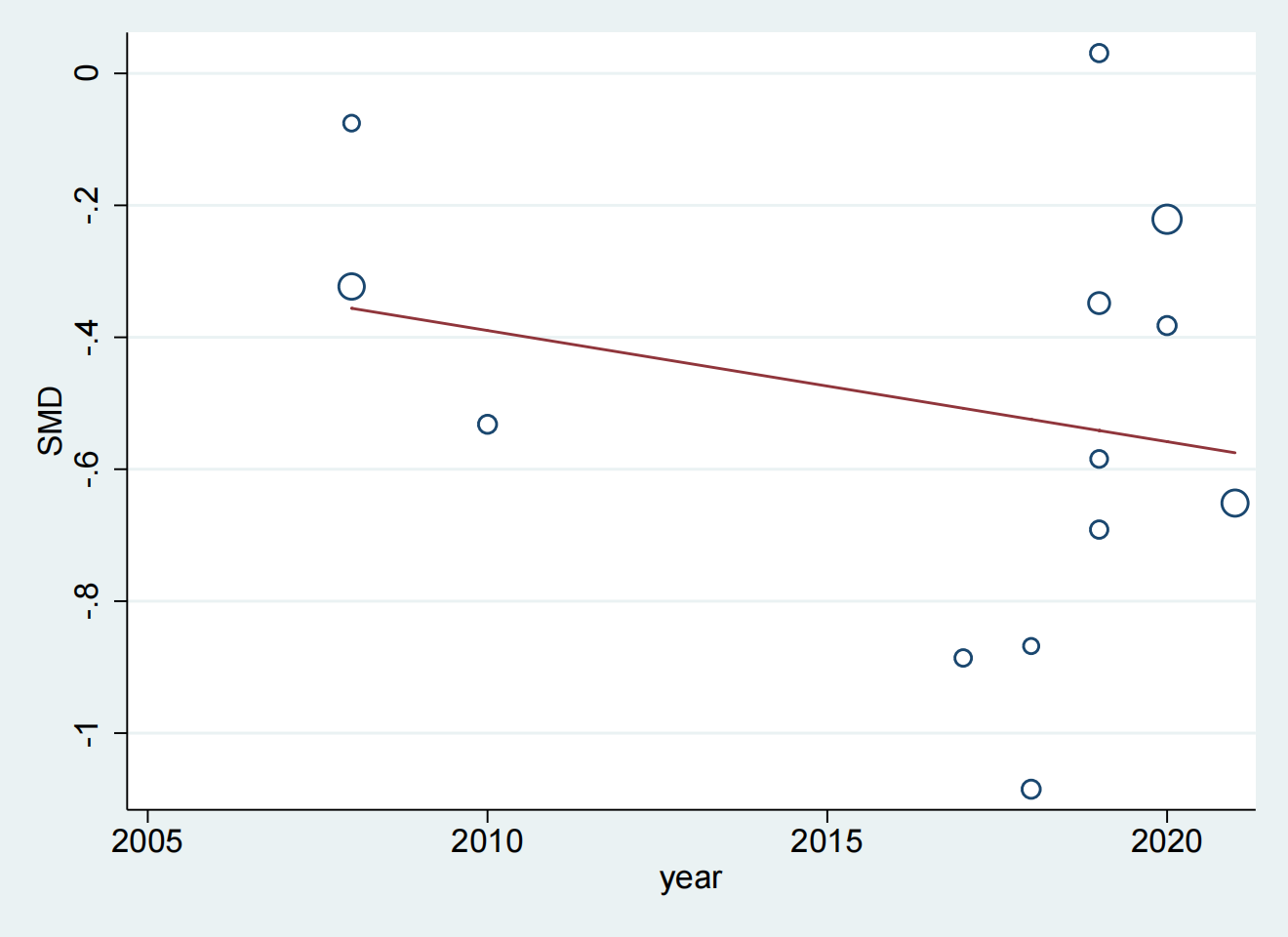
**

**Figure 9 Regression of year on FBG
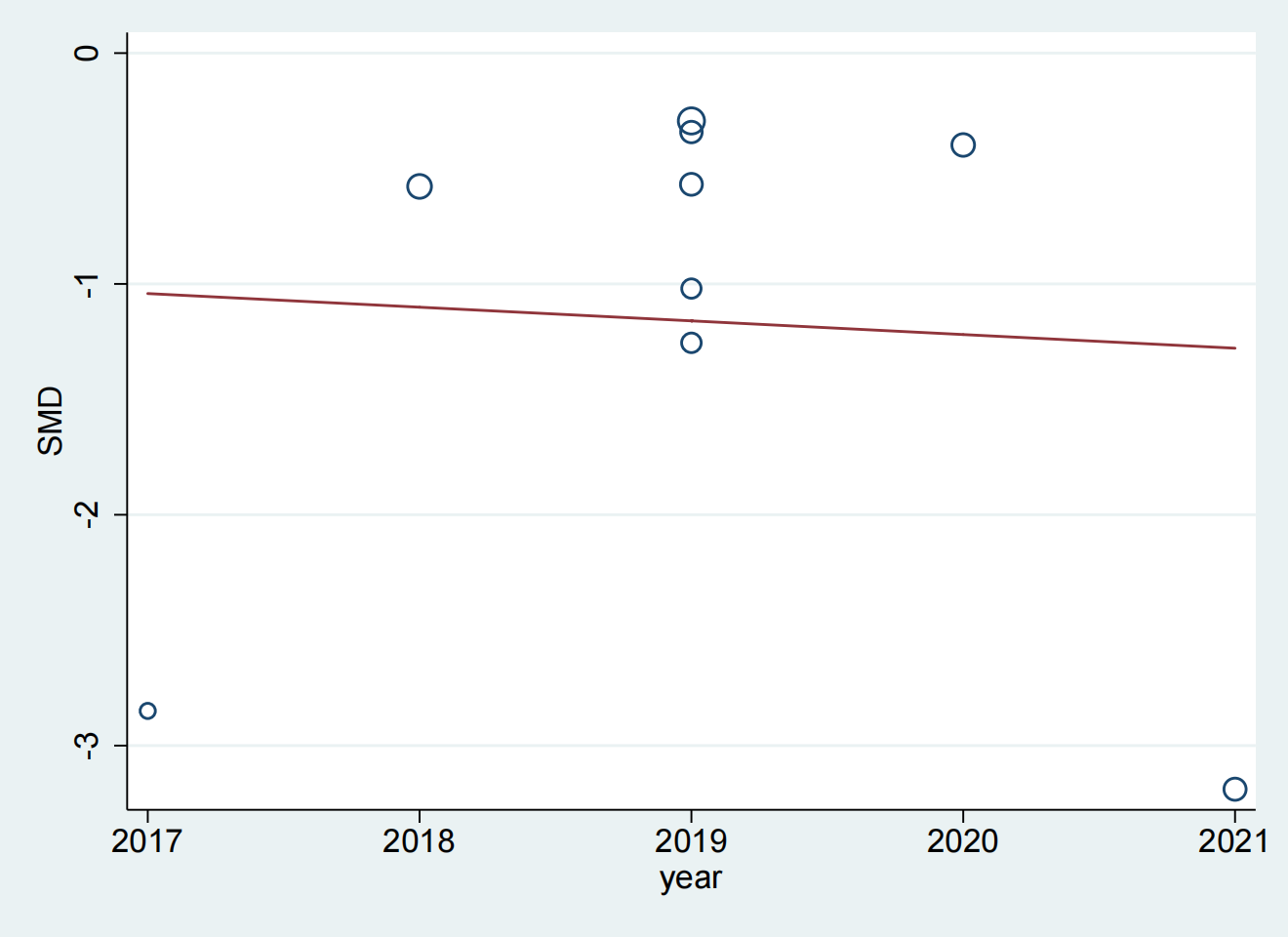
**

**Figure 10 Regression of year on HbA1c**

**
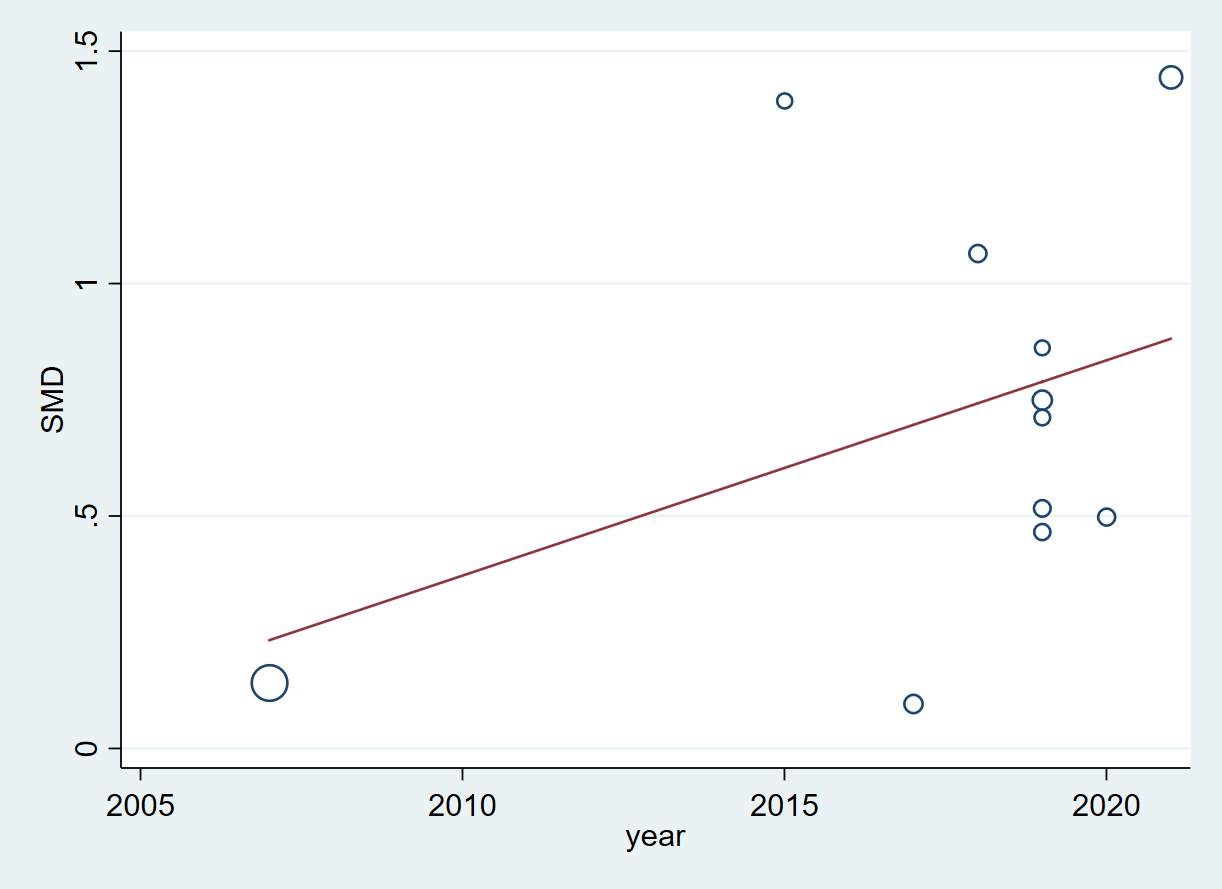
**

**Figure 11 Regression of year on HDL**

**
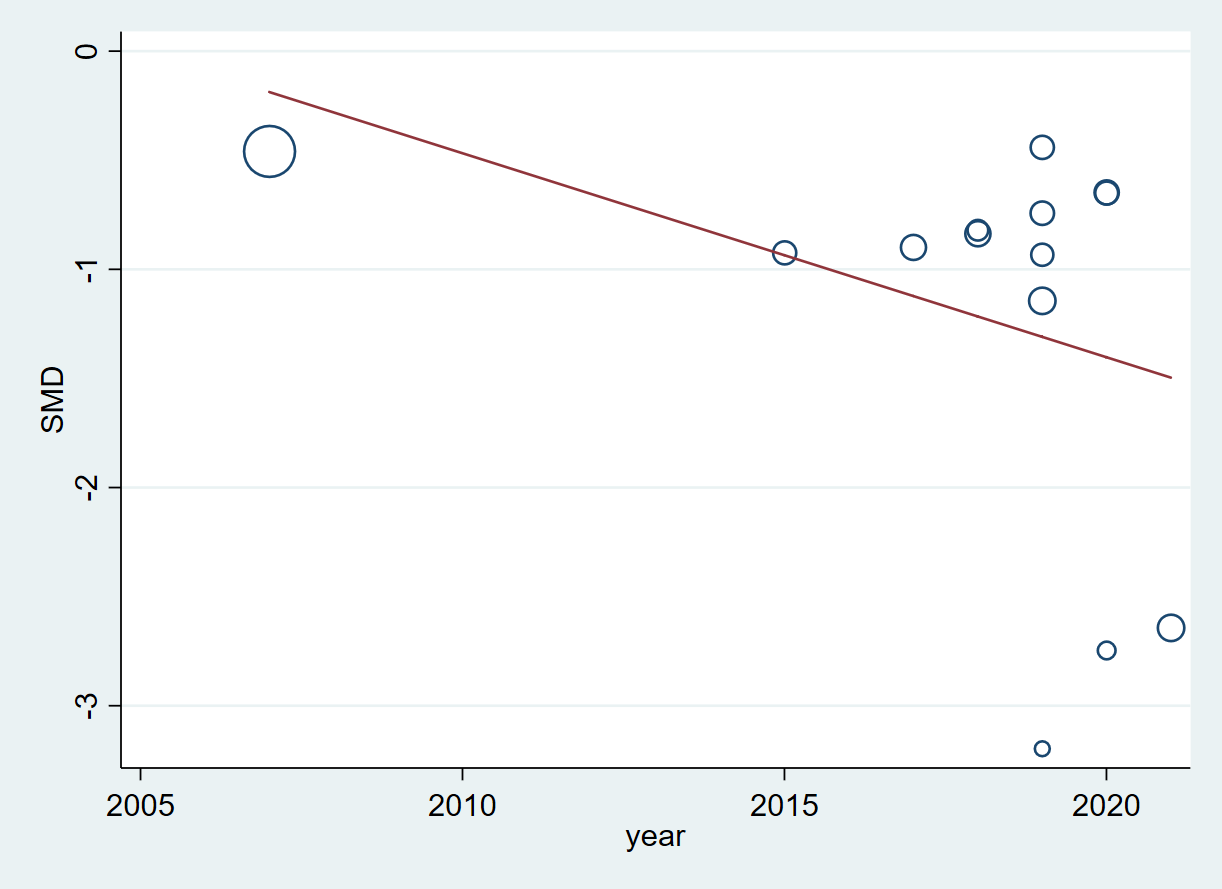
**

**Figure 12 Regression of year on LDL**

**
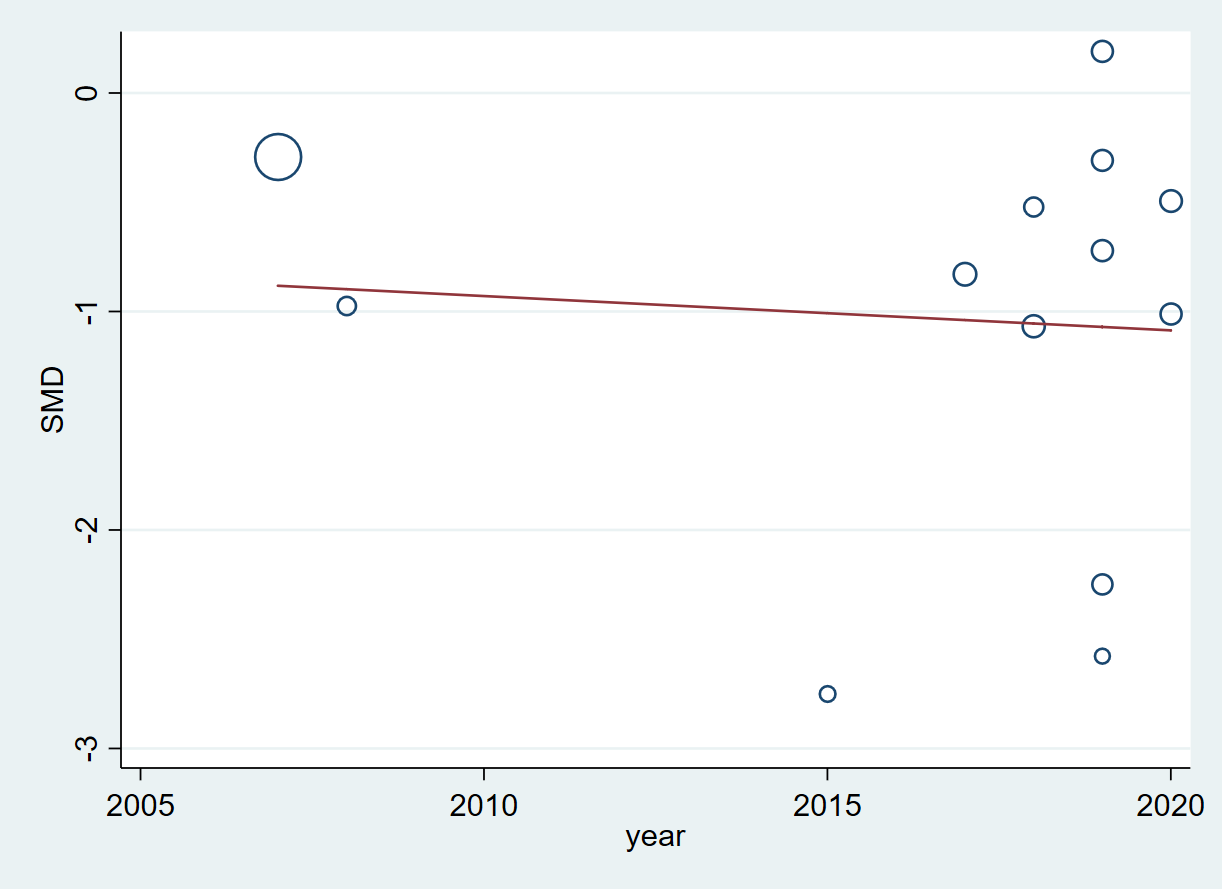
**

**Figure 13 Regression of year on TC**

**
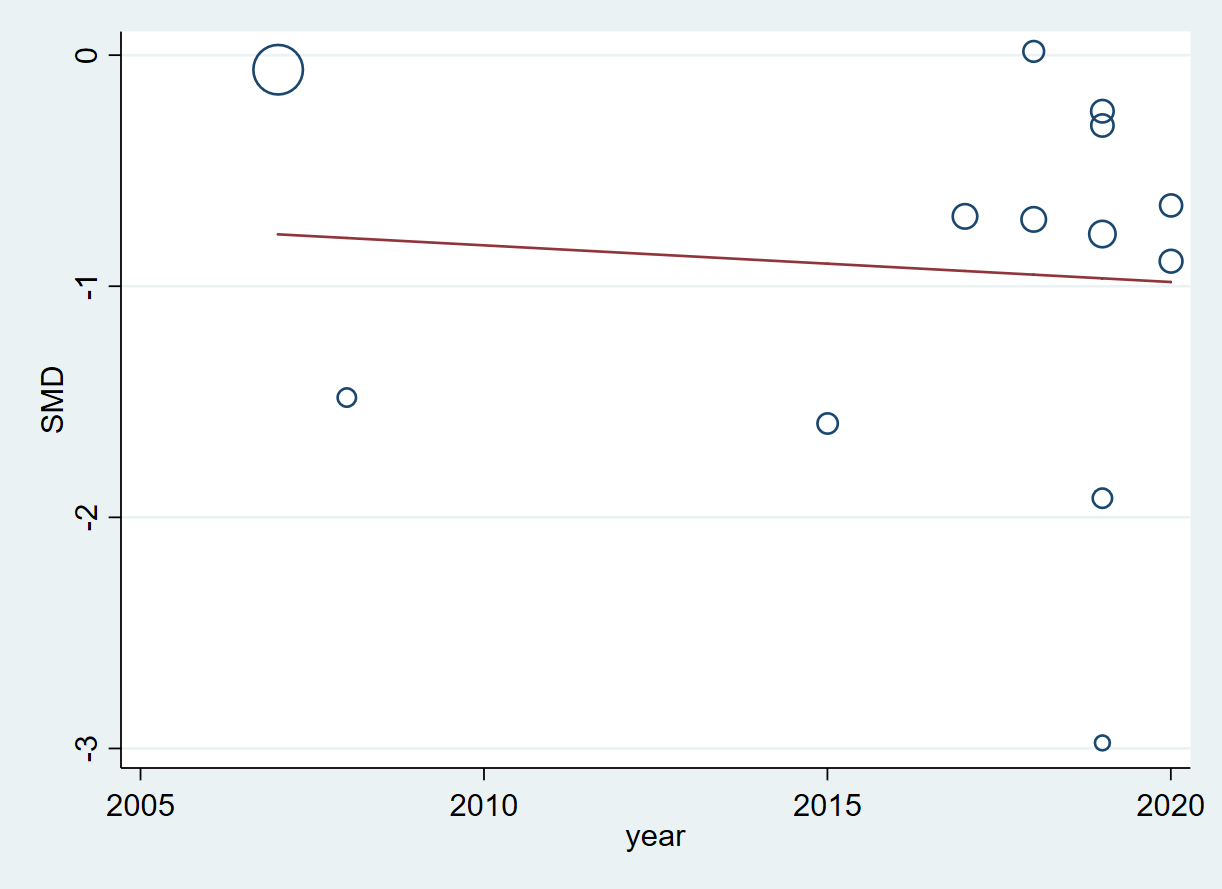
**

**Figure 14 Regression of year on TG**

### **2. Sensitivity analysis**

**
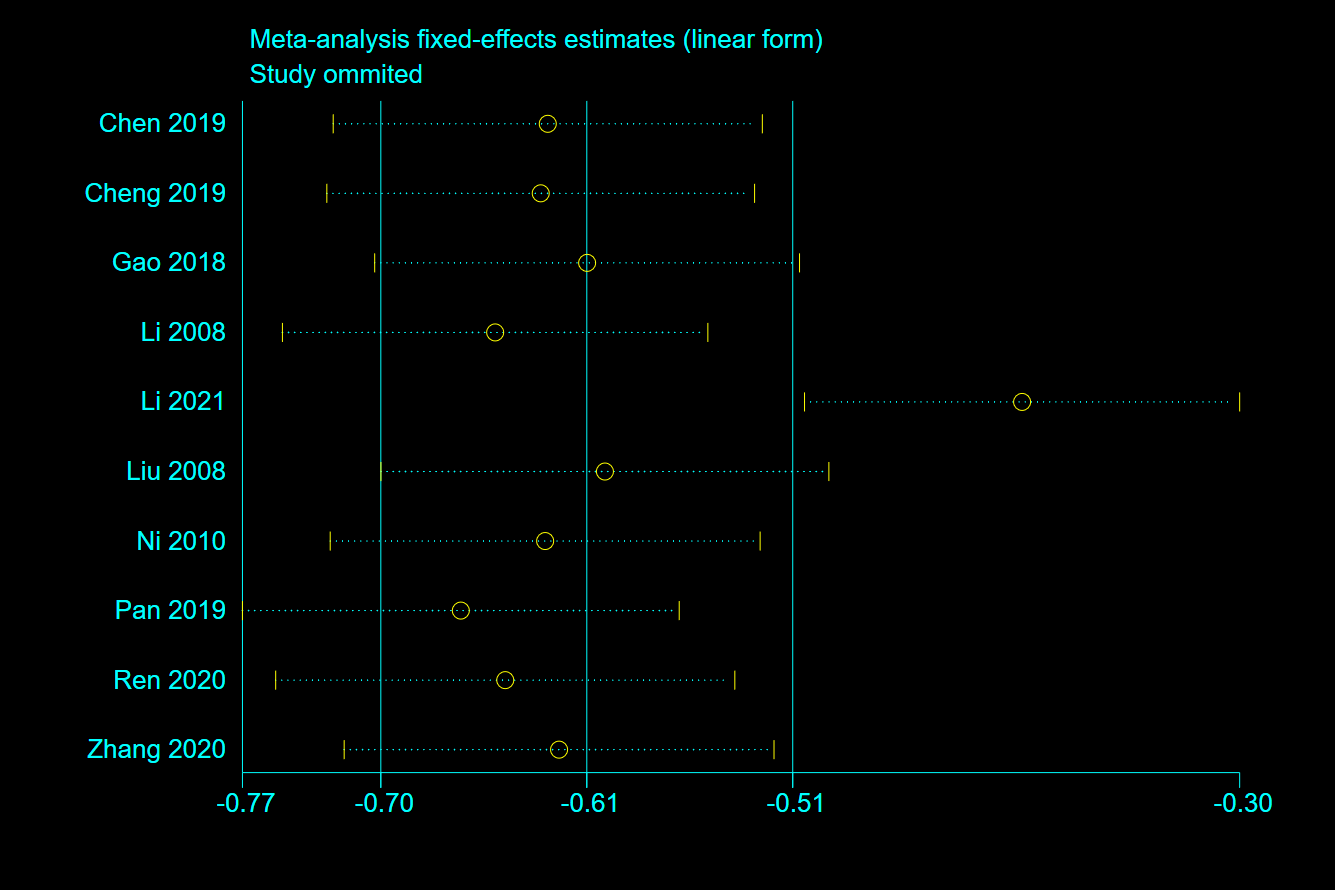
**

**Figure 15 Sensitivity analysis of TCM on 2hPG**

**
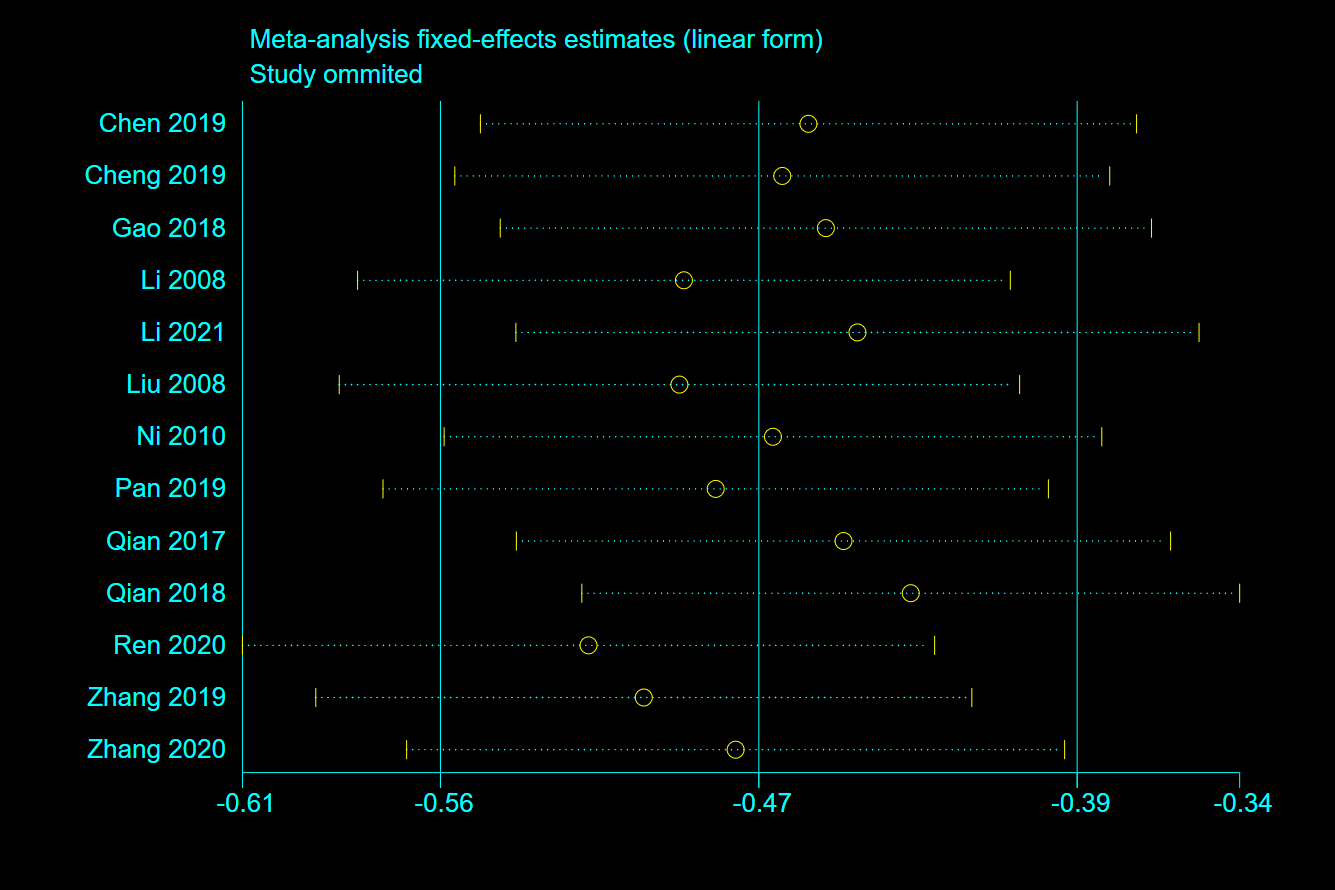
**

**Figure 16 Sensitivity analysis of TCM on FBG**

**
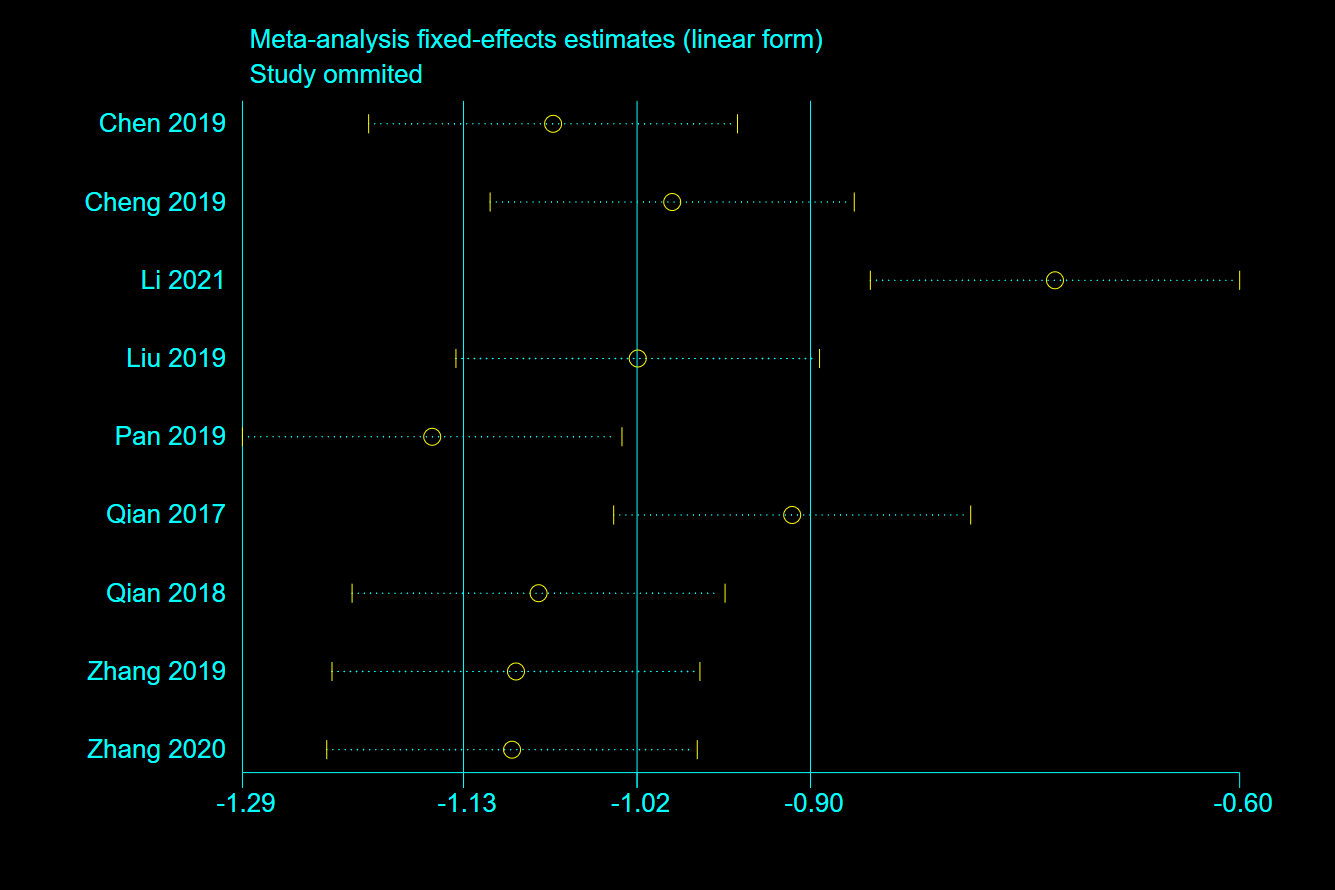
**

**Figure 17 Sensitivity analysis of TCM on HbA1c**

**
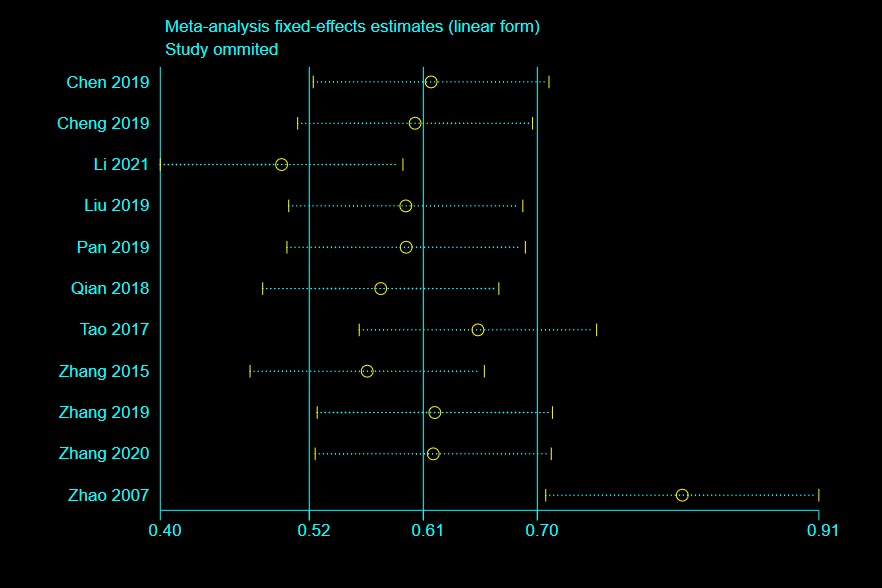
**

**Figure 18 Sensitivity analysis of TCM on HDL**

**
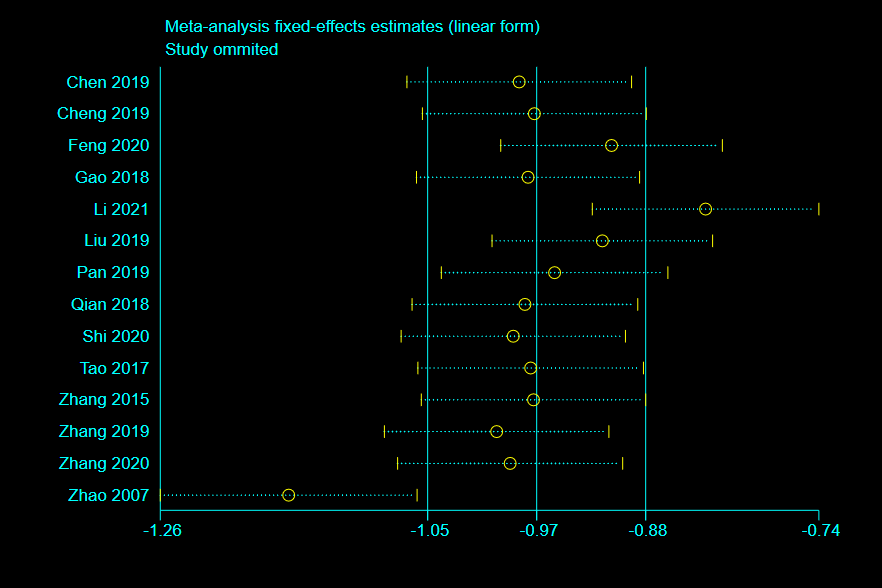
**

**Figure 19 Sensitivity analysis of TCM on LDL**

**
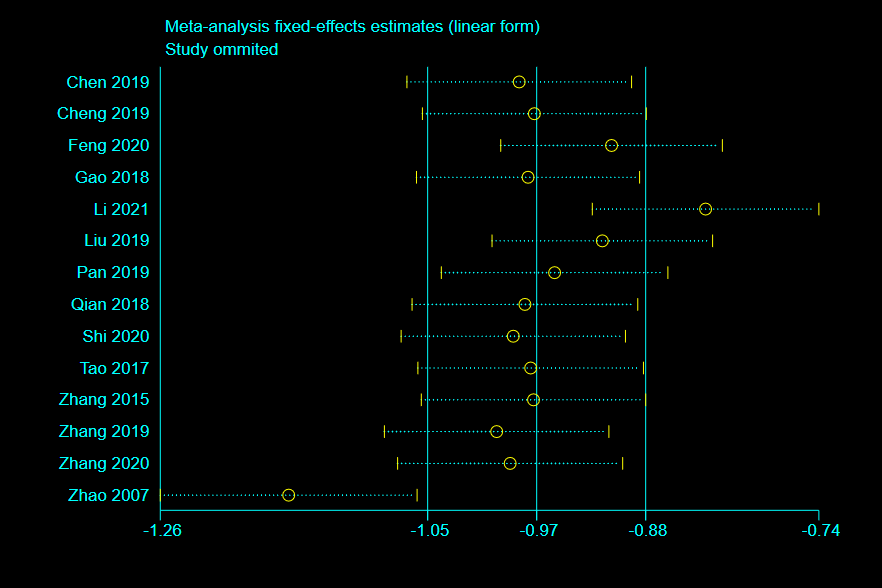
**

**Figure 20 Sensitivity analysis of TCM on TC**

**
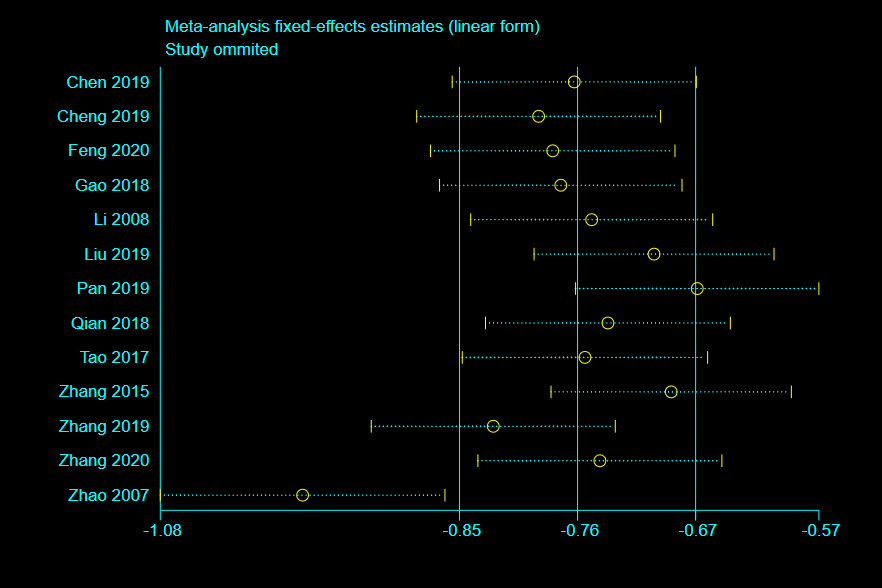
**

**Figure 21 Sensitivity analysis of TCM on TG**

**
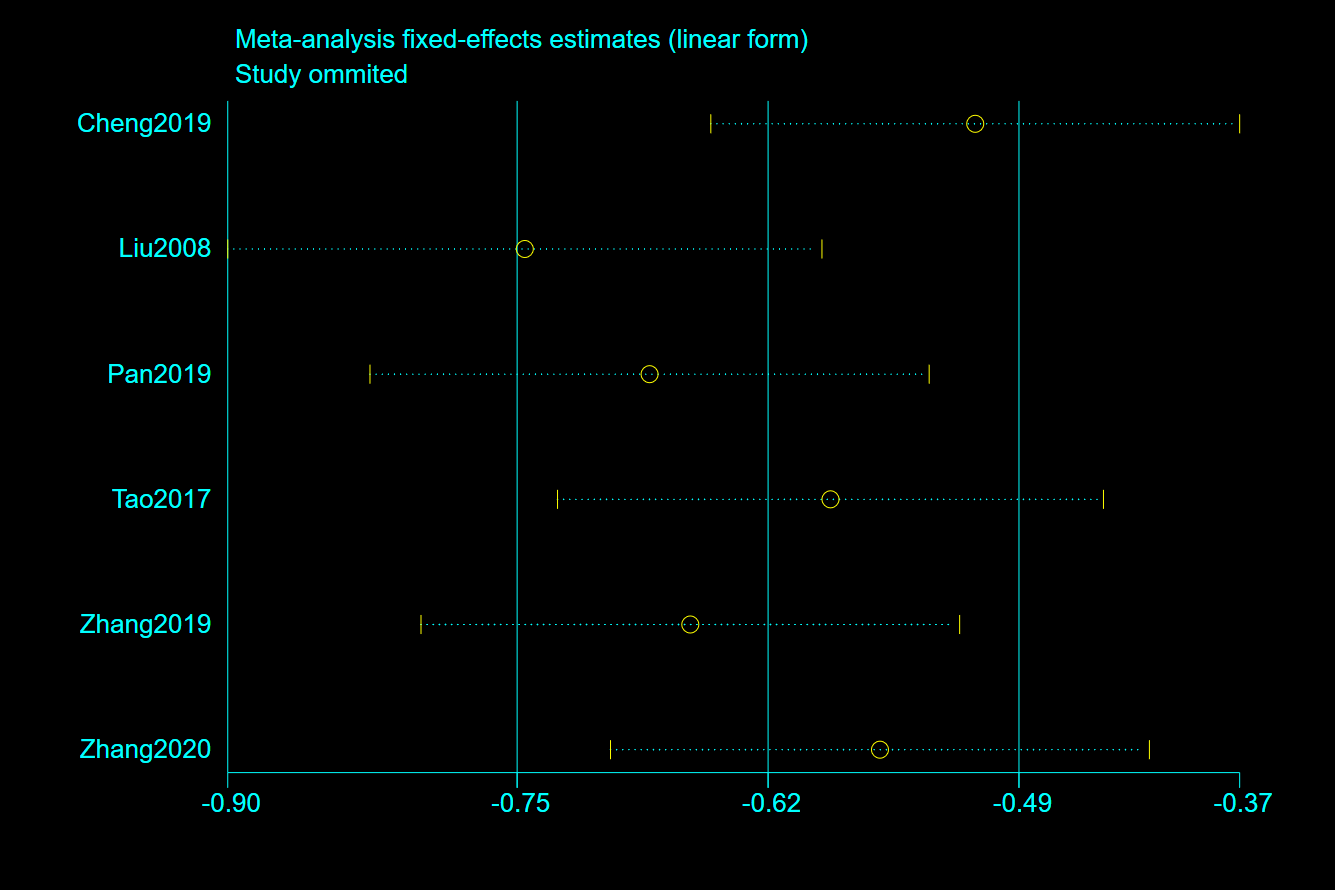
**

**Figure 22 Sensitivity analysis of TCM on CRP**

**
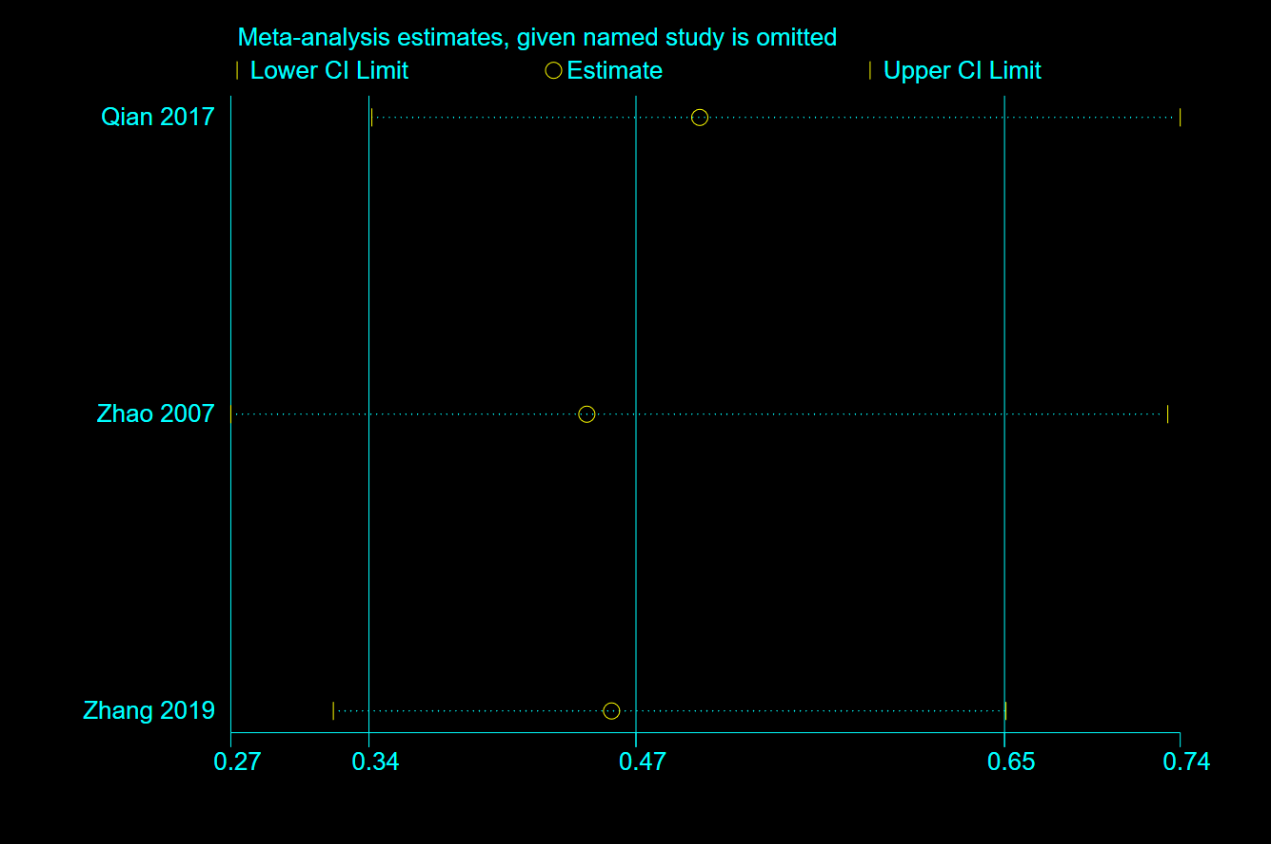
**

**Figure 23 Sensitivity analysis of TCM on cardiovascular events**

**
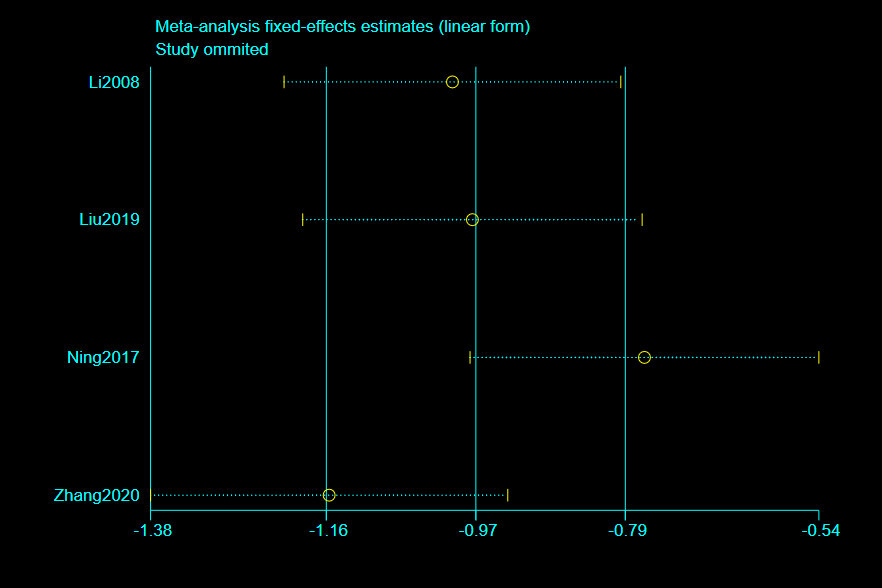
**

**Figure 24 Sensitivity analysis of TCM on FIB**

**
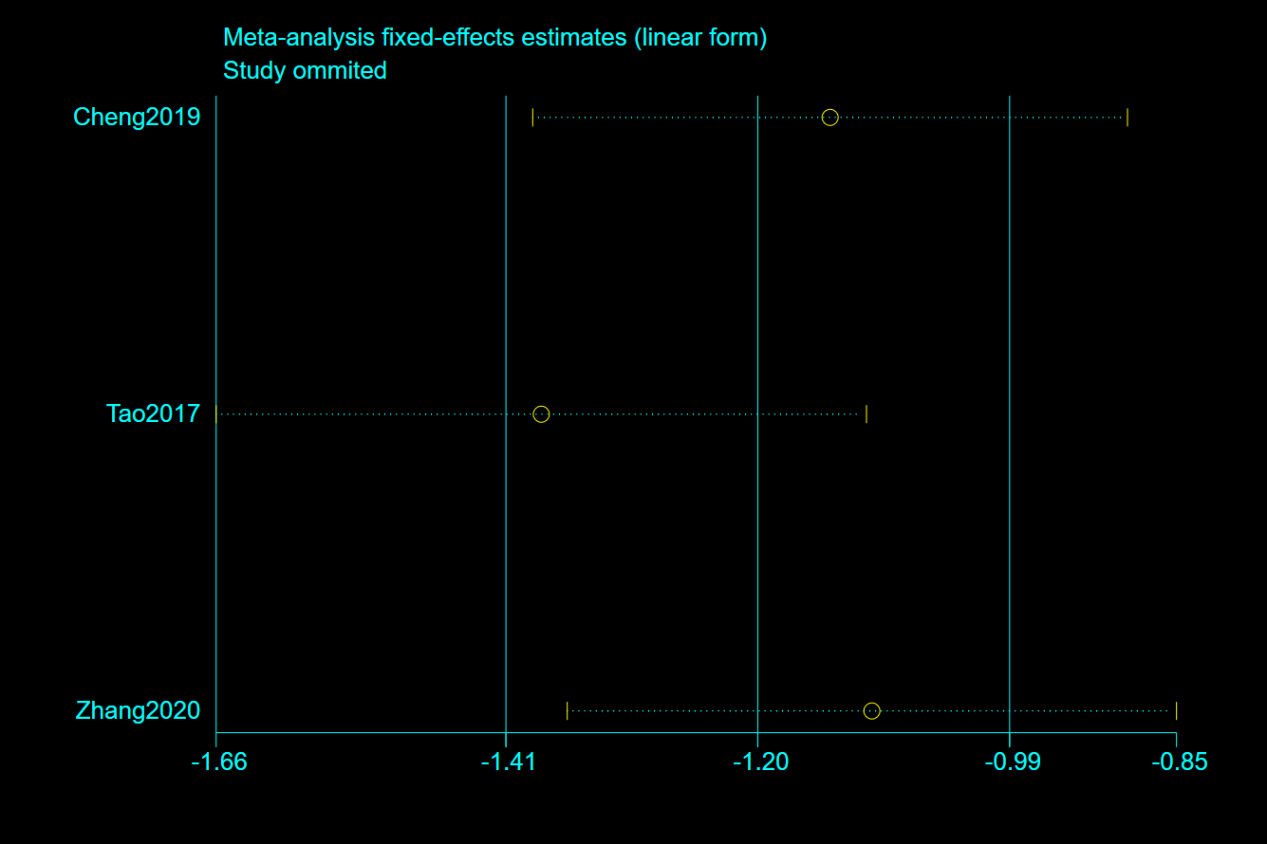
**

**Figure 25 Sensitivity analysis of TCM on IL-6**

**
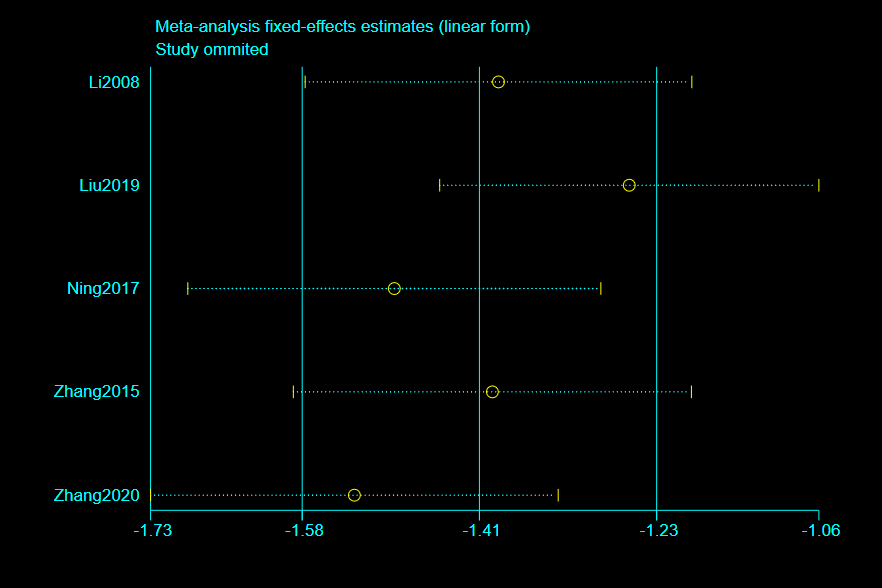
**

**Figure 26 Sensitivity analysis of TCM on plasma viscosity**

**
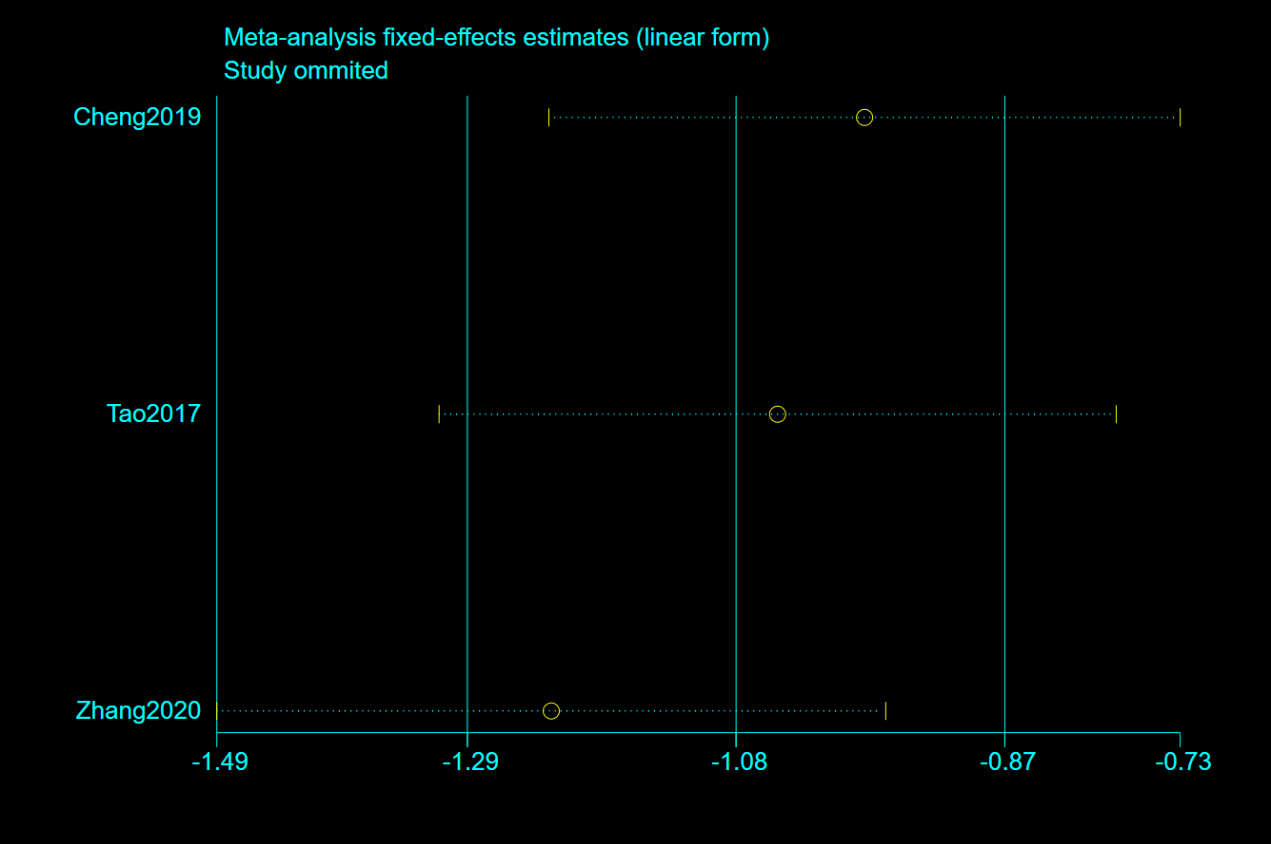
Figure 27 Sensitivity analysis of TCM on TNF-α**

**
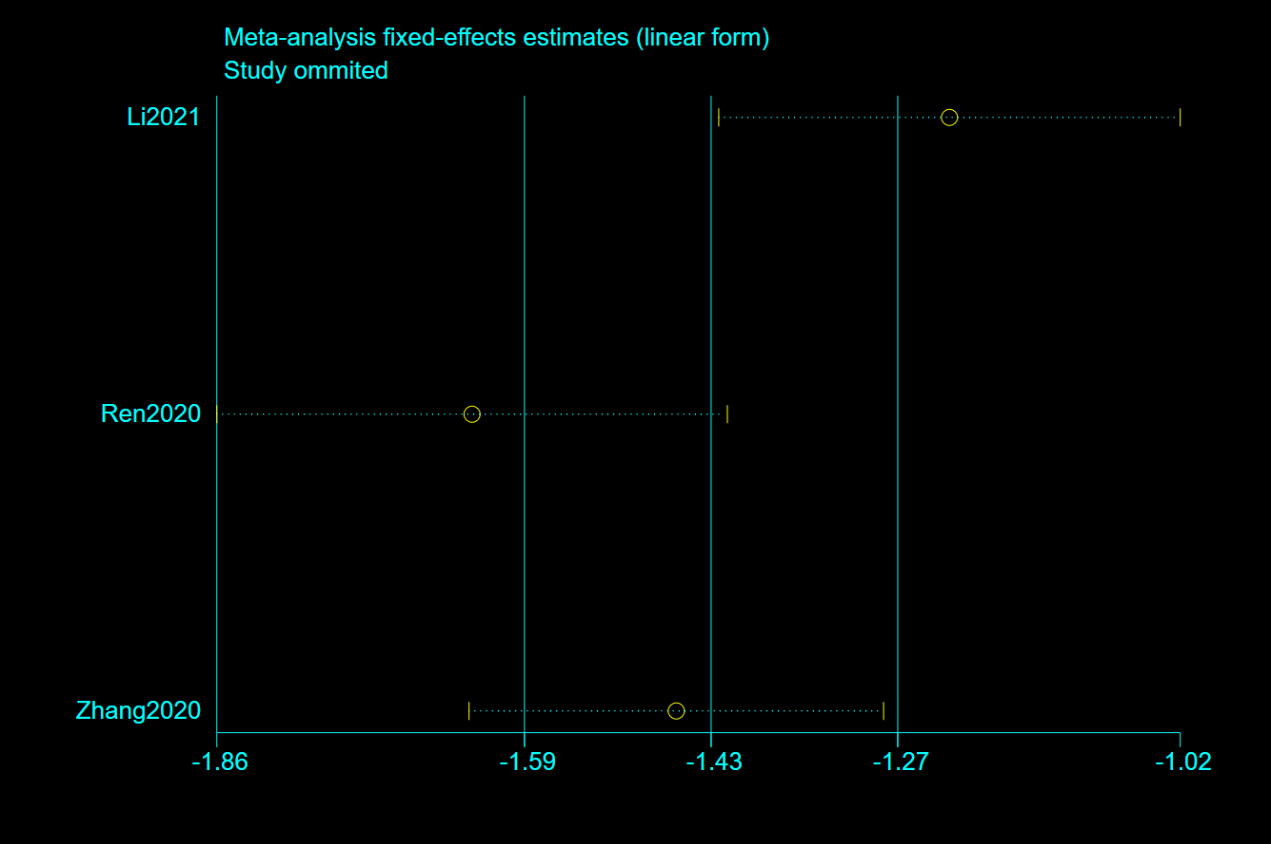
**

**Figure 28 Sensitivity analysis of TCM on LVEDD**

**
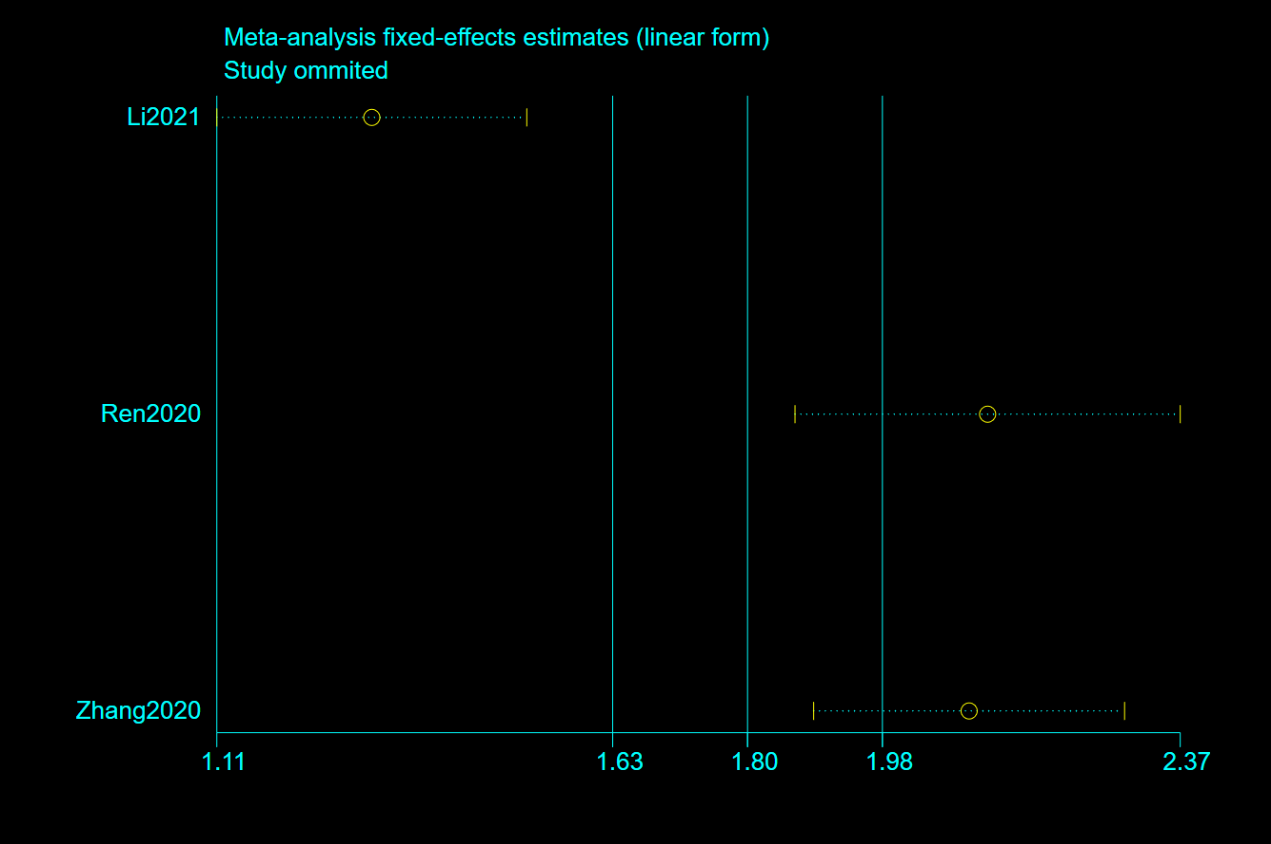
**

**Figure 29 Sensitivity analysis of TCM on LVEF**

**
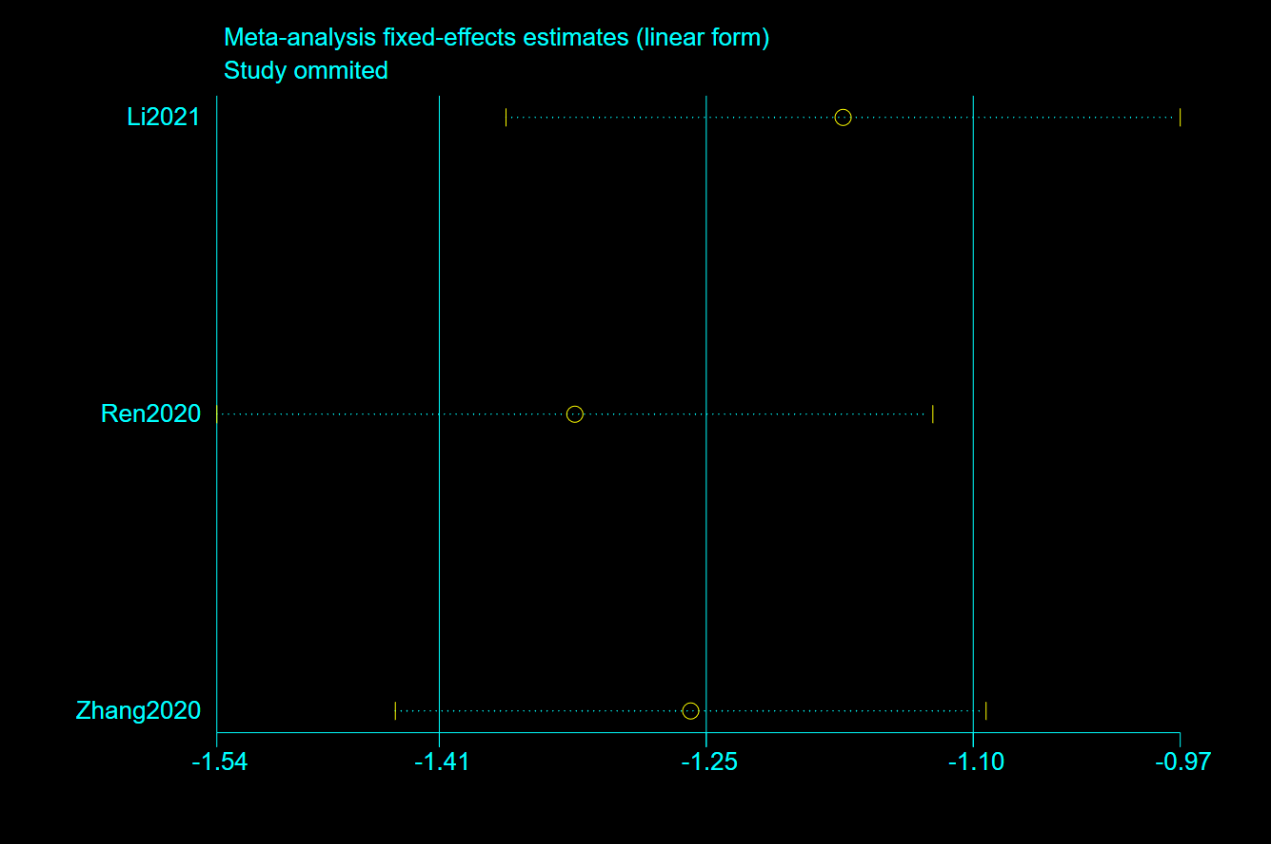
**

**Figure 30 Sensitivity analysis of TCM on LVESD**

1. **Publication Bias**

**3.1The funnel plot and Edger's test results for TC**

**Egger's test**

| Std_Eff \| Coef. Std. Err. t P>\|t\| [95% Conf. Interval] |
| --- |
| slope \| -.7507727 .4077628 -1.84 0.093 -1.648253 .1467072 |
| bias \| -.6133013 3.301318 -0.19 0.856 -7.879454 6.652851 |


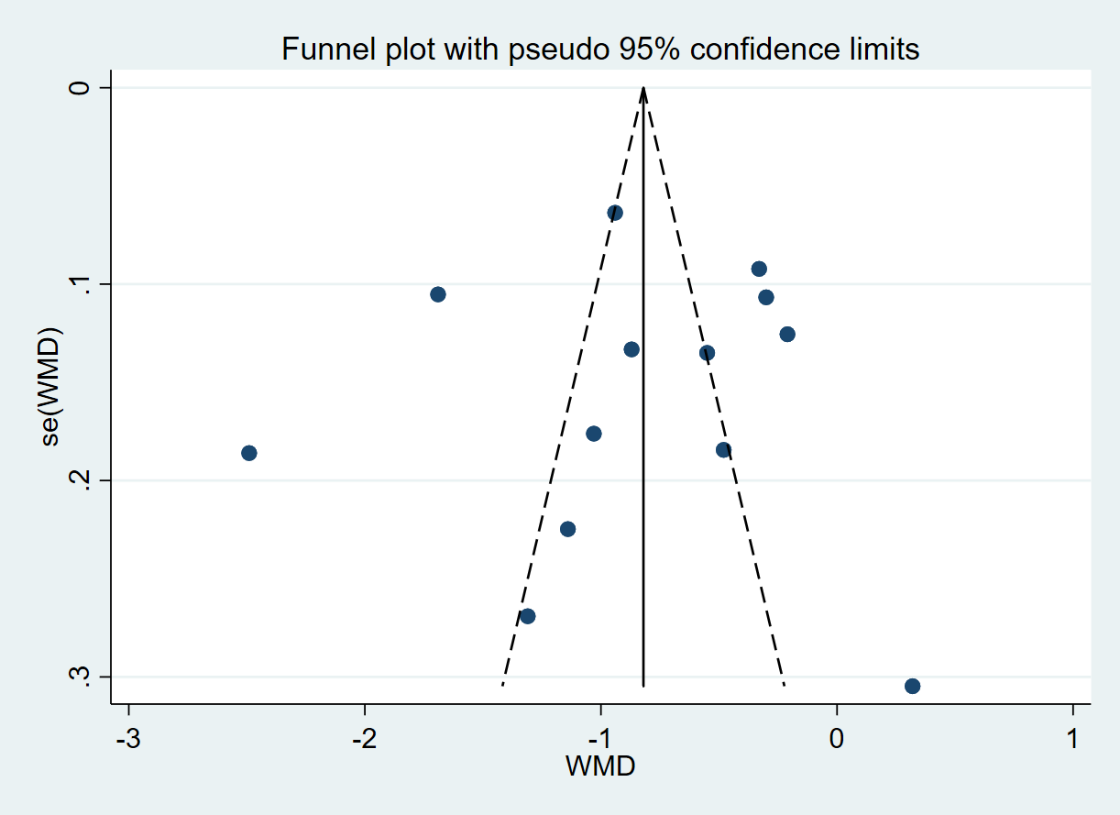


**3.2 The funnel plot and Edger's test results for TG**

**Egger's test**

| Std_Eff | Coef. | Std. | Err. | t | P>\|t\| | [95%Conf.Interval] |
| --- | --- | --- | --- | --- | --- | --- |
| slope | -0.6215302 | 0.197387 | -3.15 | 0.009 | -1.055976 | -0.1870844 |
| bias | 0.924412 | 2.17084 | 0.43 | 0.678 | -3.853574 | 5.702398 |


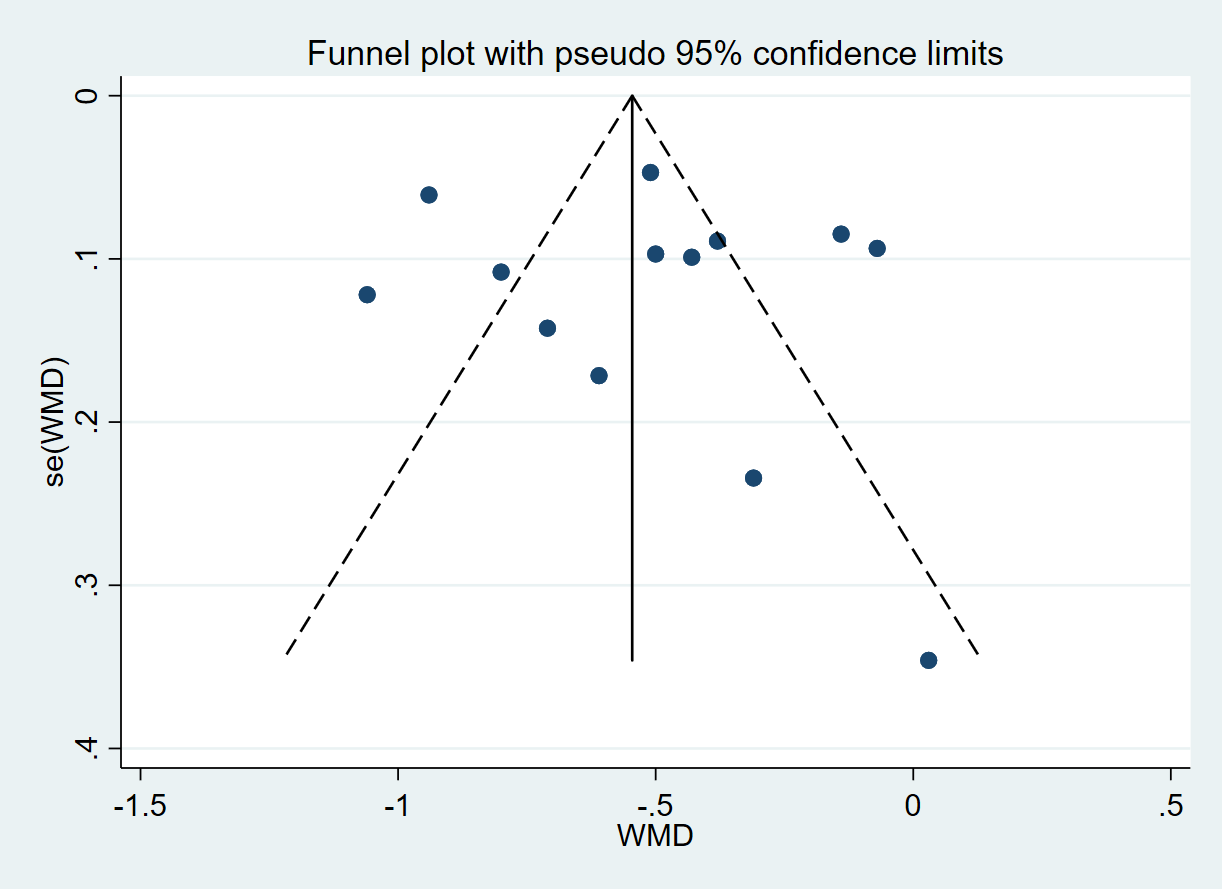


**3.3 The funnel plot and Edger's test results for LDL**

**Egger's test**

| Std_Eff | Coef. | Std. | Err. | t | P>\|t\| | [95%Conf.Interval] |
| --- | --- | --- | --- | --- | --- | --- |
| slope | -0.645742 | 0.2406551 | -2.68 | 0.02 | -1.170084 | -0.1213995 |
| bias | -0.2466274 | 3.052826 | -0.08 | 0.937 | -6.898163 | 6.404908 |


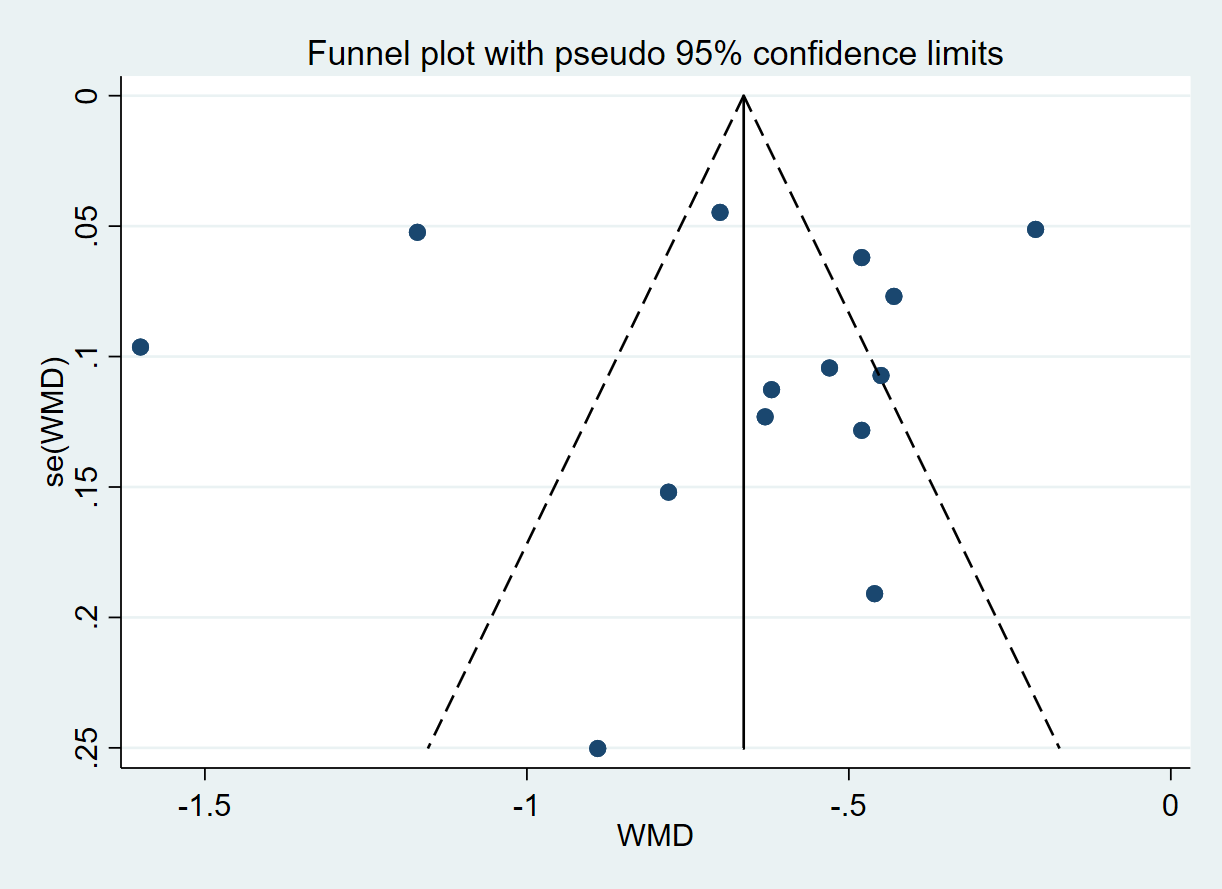


**3.4 The funnel plot and Edger's test results for HDL**

**Egger's test**

| Std_Eff | Std_Eff | Coef. | Std. | Err. | t | P>\|t\| | [95%Conf.Interval] |
| --- | --- | --- | --- | --- | --- | --- | --- |
| slope | slope | 0.1419133 | 0.114444 | 1.24 | 0.246 | -0.116977 | 0.4008036 |
| bias | bias | 2.210332 | 2.174865 | 1.02 | 0.336 | -2.709554 | 7.130219 |


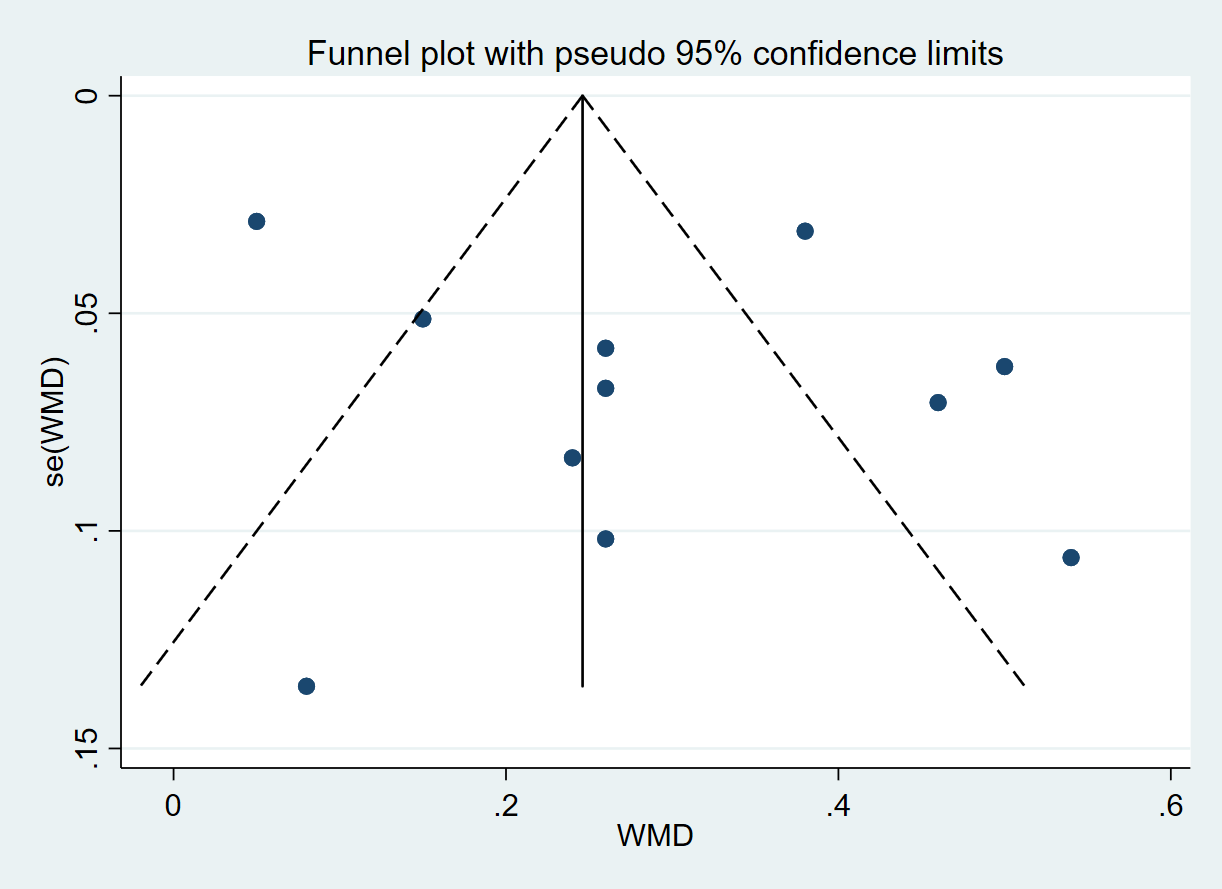


**3.5 The funnel plot and Edger's test results for 2h-PG**

**Egger's test**

| **Std_Eff** | **Coef.** | **Std. Err.** | **t** | **P>\|t\|** | **[95% Conf. Interval]** | |
| --- | --- | --- | --- | --- | --- | --- |
| **slope** | -1.106906 | 0.7779802 | -1.42 | 0.193 | -2.900932 | 0.6871195 |
| **bias** | 0.7304332 | 3.88316 | 0.19 | 0.855 | -8.22415 | 9.685017 |


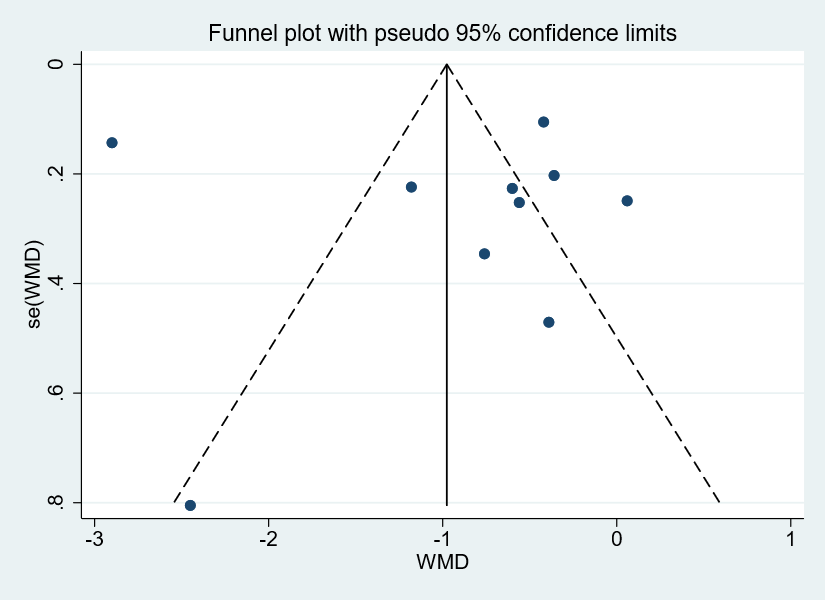

Supplement: Supplementary Materials — The metaregression analysis of sample, publication year, and sensitivity analysis are available in supplementary materials. [file 2545476.f1.docx]
